# Innovative Solutions for High-Performance Silicon Anodes in Lithium-Ion Batteries: Overcoming Challenges and Real-World Applications

**DOI:** 10.1007/s40820-024-01388-3

**Published:** 2024-04-24

**Authors:** Mustafa Khan, Suxia Yan, Mujahid Ali, Faisal Mahmood, Yang Zheng, Guochun Li, Junfeng Liu, Xiaohui Song, Yong Wang

**Affiliations:** 1 Institute for Energy Research, Jiangsu University, Zhenjiang, 212013 Jiangsu People’s Republic of China; 2 School of Energy and Power Engineering, Jiangsu University, Zhenjiang, 212013 Jiangsu People’s Republic of China; 3 School of Materials Science and Engineering, Hefei University of Technology, Hefei, 230009 Anhui People’s Republic of China

**Keywords:** Silicon anode, Energy storage, Nanostructure, Prelithiation, Binder

## Abstract

Silicon (Si) has emerged as a potent anode material for lithium-ion batteries (LIBs), but faces challenges like low electrical conductivity and significant volume changes during lithiation/delithiation, leading to material pulverization and capacity degradation. Recent research on nanostructured Si aims to mitigate volume expansion and enhance electrochemical performance, yet still grapples with issues like pulverization, unstable solid electrolyte interface (SEI) growth, and interparticle resistance. This review delves into innovative strategies for optimizing Si anodes’ electrochemical performance via structural engineering, focusing on the synthesis of Si/C composites, engineering multidimensional nanostructures, and applying non-carbonaceous coatings. Forming a stable SEI is vital to prevent electrolyte decomposition and enhance Li^+^ transport, thereby stabilizing the Si anode interface and boosting cycling Coulombic efficiency. We also examine groundbreaking advancements such as self-healing polymers and advanced prelithiation methods to improve initial Coulombic efficiency and combat capacity loss. Our review uniquely provides a detailed examination of these strategies in real-world applications, moving beyond theoretical discussions. It offers a critical analysis of these approaches in terms of performance enhancement, scalability, and commercial feasibility. In conclusion, this review presents a comprehensive view and a forward-looking perspective on designing robust, high-performance Si-based anodes the next generation of LIBs.

## Highlights

- *Si/C Composite and Nanostructure Engineering*: Advanced Si/C composites and multidimensional nanostructures address key challenges in silicon anodes, like volume expansion and unstable SEI, enhancing LIBs performance.
- *Artificial SEI, Prelithiation, and Binders*: Focus on stable artificial SEI layers, efficient prelithiation, and cutting-edge binders to improve Coulombic efficiency and reduce capacity loss, enhancing Si anode durability and efficiency.
- *Real-World Application and Scalability*: Analysis of these strategies highlights scalability and commercial viability, transitioning Si-anode technologies to practical, high-performance LIBs applications.

## Graphical abstract

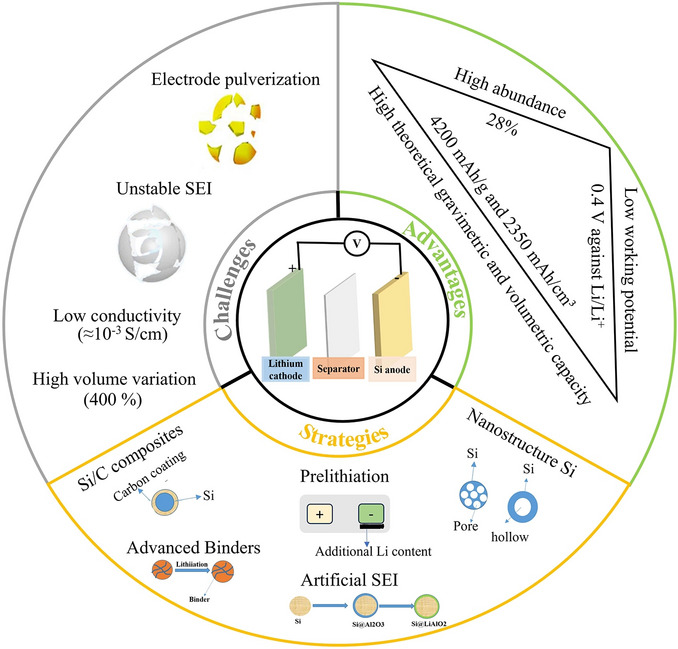

## Introduction

With its significant theoretical capacity and affordable cost [1–4], the lithium-ion batteries (LIBs) have emerged as an ideal candidate to meet the escalating demand for electric vehicles. This demand encompasses a variety of requirements: high energy density for extended driving range, high power density for efficient acceleration, lightweight for optimal vehicle performance, and rapid charging for user convenience. While LIBs have demonstrated exceptional potential for powering portable electronic devices, they are also showing great promise for use in electric and hybrid vehicles. However, contemporary commercial LIBs possess somewhat limited volumetric and gravimetric energy densities, making it challenging to satisfy the growing needs of grid-energy storage and electric vehicles. Though the cycle life of LIBs in small-scale energy storage under consistent conditions is well-documented, their electrochemical performance in large-scale energy storage, particularly under the fluctuating conditions typical of renewable energy, remains an area requiring further exploration.

The performance metrics of LIBs, encompassing energy density, operational longevity, and safety, hinge largely upon their core functional components, most notably the electrode materials. Current commercial LIBs predominantly employ metal oxides or phosphate cathodes and graphite-based anodes. Given that these commercial graphite anodes possess an added theoretical capacity limited to 372 mAh g^−1^ [5, 6], there is an urgent need for anode materials with higher capacities. This is paramount for fulfilling the aspirations of more energy-dense, cost-effective LIBs, thereby catalyzing the evolution of electric vehicles. In this realm, lithium-alloying reactions, as opposed to intercalation processes, present an avenue for attaining elevated capacities due to the increased storage potential of lithium ions within a host material during the alloying process. Among the various alloying materials proposed for LIB anodes, such as Si, Pb, Ga, In, Ag, Ge, and Mg [7–13], silicon (Si) stands out. It boasts an unparalleled theoretical capacity of 4200 mAh g^−1^ coupled with a low operational potential, solidifying its credentials as a desirable anode material for LIBs [14–18]. Nonetheless, challenges persist: the Si anode’s susceptibility to fracture during the alloying/dealloying stages leads to issues like pulverization and delamination from the collector [19, 20]. Consequently, this fosters the formation of an unstable solid electrolyte interphase (SEI) on the Si surface, entrapping lithium, and culminating in irreversible capacity degradation and subpar initial Coulombic efficiency (ICE). Moreover, factors such as lithium’s sluggish diffusion in Si and Si's inherent low electrical conductivity adversely influence the capacity utilization and rate capabilities of Si electrodes.

To address these obstacles, various methodologies have been conceptualized. Central to this endeavor is the design and synthesis of Si-centric materials with tailored architectures. This work comprehensively examines the lithiation mechanism and challenges associated with Si-based anodes, proposing solutions such as Si/C composites, nanostructured Si-based materials, Si derivatives, and non-carbonaceous materials specifically designed for Si-based anodes. We delve into influencing factors such as binder effects and prelithiation strategies. Additionally, the concept of a stable artificial SEI is explored to prevent electrolyte infiltration and decomposition, addressing the issue of low CE caused by the unstable natural SEI film.

Recent advancements in the field have introduced innovative approaches to Si/C composites, nanostructured Si materials, and SiO_*x*_/C type composites. These developments focus on nanoengineering techniques that enhance performance by overcoming issues like significant volume expansion and poor cycling performance. For instance, the interfacial incorporation of metal nanocrystals in Si anodes has been shown to boost the ICE by improving electrical conductivity and reducing interparticle resistance [21]. This satellite-like architecture offers a new perspective on addressing the challenges of low Coulombic Efficiency and unstable SEI formation.

Emerging trends in structural engineering of Si nanomaterials involve the integration with conductive carbons, metals, and metal oxides. These enhancements in the electrochemical performance of Si-based anodes are crucial in overcoming the pronounced volume changes and unstable SEI formation. The engineered design of artificial SEI layers and the impact of prelithiation processes and advanced binder systems are pivotal in enhancing the performance and stability of Si-based anodes [22].

**Fig. 1:**
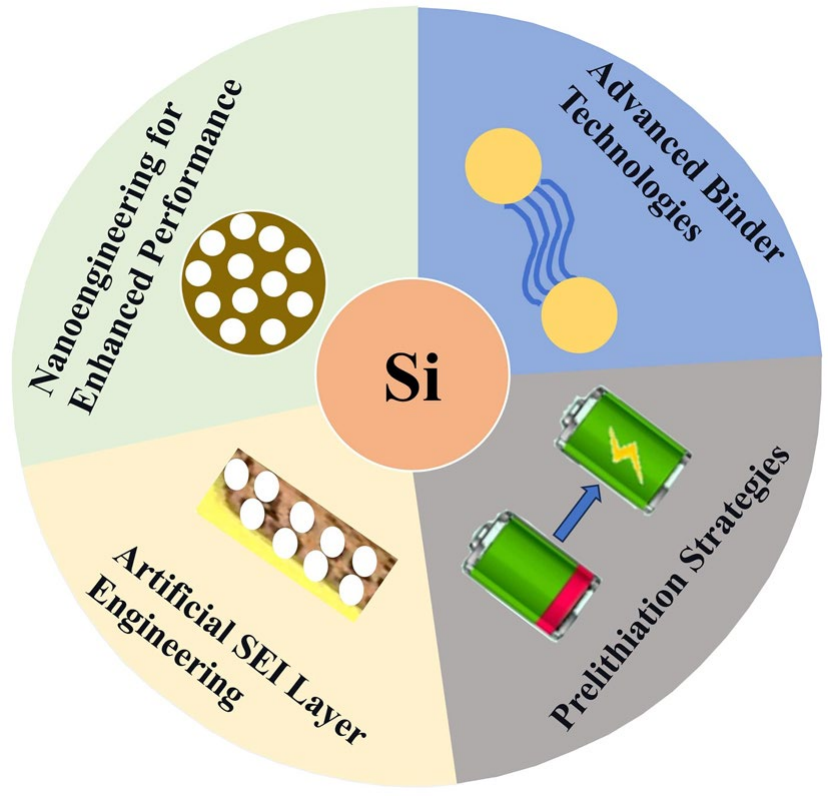
A schematic diagram illustrating the strategies for addressing the multifaceted challenges inherent to Si-based anodes in LIBs

Our research contributes to this evolving field by addressing unresolved challenges and opening new avenues for exploration. We provide novel insights into the optimized use of Si-based materials, potentially offering solutions to enhance the practical application of Si anodes in LIBs. This work aligns with the latest developments and aims to further the understanding and application of Si-based anode materials. We provide a forward-looking perspective by identifying gaps in current research and suggesting potential future directions. This aspect of our review adds significant value by guiding researchers and industry professionals toward the future trajectory of Si-based anode materials, thereby contributing to the novelty of our review and setting it apart from previous publications in the field. Our work aims to offer a comprehensive, up-to-date, and application-oriented overview of the current state and future prospects of Si-based anodes in LIB technology. Figure 1 provides a schematic overview, illustrating the prospective strategies pertinent to the utilization of Si-based anodes in LIBs, while Table 1 details the electrochemical properties of various materials used as anodes in LIBs.

**Table 1:** Electrochemical properties of several materials used as anode in LIBs

Anode material	Theoretical gravimetric capacity (mAh g^−1^)	Working potential (V)	Volume variation (%)
Si	4200	0.4	400
Li	3862	0	100
Ge	1625	0.5	370
Sn	994	0.6	26
Graphite	372	0.05	12
Li_4_Ti_5_O_12_	175	1.5	1
TiO_2_	167	0.8	< 4

### Electrochemical Lithiation Mechanism and Challenges of the Si Anode

The electrochemical behavior of Si has been extensively studied, leading to the formation of various compounds including LiSi, Li_12_Si_7_, Li_13_Si_4_, Li_15_Si_4_, and Li_22_Si_5_ [23]. The properties of these compounds are detailed in Table 2 [24]. The lithiation mechanism of Si, which is essential for enhancing its electrochemical performance, has been a focal point for many researchers [25–27]. XRD analysis was employed to understand phase transformations [28, 29], revealing the following reaction mechanism:

**Table 2:** Theoretical capacity and volume variation of different phases of Li–Si

Phase	Theoretical capacity (mAh g^−1^)	Volume variation (%)
Si	0	0
LiSi	954	160
Li_12_Si_7_	1635	222
Li_2_Si	1900	263
Li_13_Si_4_	3100	389
Li_15_Si_4_	3590	390
Li_22_Si_5_	4200	400

Discharging process:

1 $${\text{Si}}\left({\text{crystalline}}\right)+x{{\text{Li}}}^{+}+x{e}^{-}\to {{\text{Li}}}_{x}{\text{Si}}\left({\text{amorphous}}\right)+\left(3.75-x\right){{\text{Li}}}^{+}+\left(3.75-x\right){e}^{-}$$

2 $$\to {{\text{Li}}}_{15}{{\text{Si}}}_{4}\left({\text{crystalline}}\right)$$

Charging process:

3 $${{\text{Li}}}_{15}{{\text{Si}}}_{4}\left({\text{crystalline}}\right)\to {\text{Si}}\left({\text{amorphous}}\right)+y{{\text{Li}}}^{+}+y{e}^{-}+{{\text{Li}}}_{15}{{\text{Si}}}_{4}\left({\text{residual}}\right)$$

During the initial lithiation, crystalline Si transitions to an amorphous state (1). Subsequently, Li_*x*_Si (amorphous) crystallizes into the Li_15_Si_4_ phase (2). During the first delithiation, a secondary two-phase region forms, ultimately yielding amorphous Si (3). Additionally, some residual Li_15_Si_4_ remains, which can be reduced if the Si anode's potential remains above 70 mV during cycling. In the second cycle, the two-phase region dissipates as Li ions interact with amorphous Si, producing sloping voltage plateaus indicative of a singular phase zone. Post this cycle, processes (2) and (3) reoccur, exhibiting the previously described characteristics, and reversible capacity rapidly decreases. While Si boasts a high theoretical capacity, its cycling performance is subpar, highlighting the need for further improvements.

**Fig. 2:**
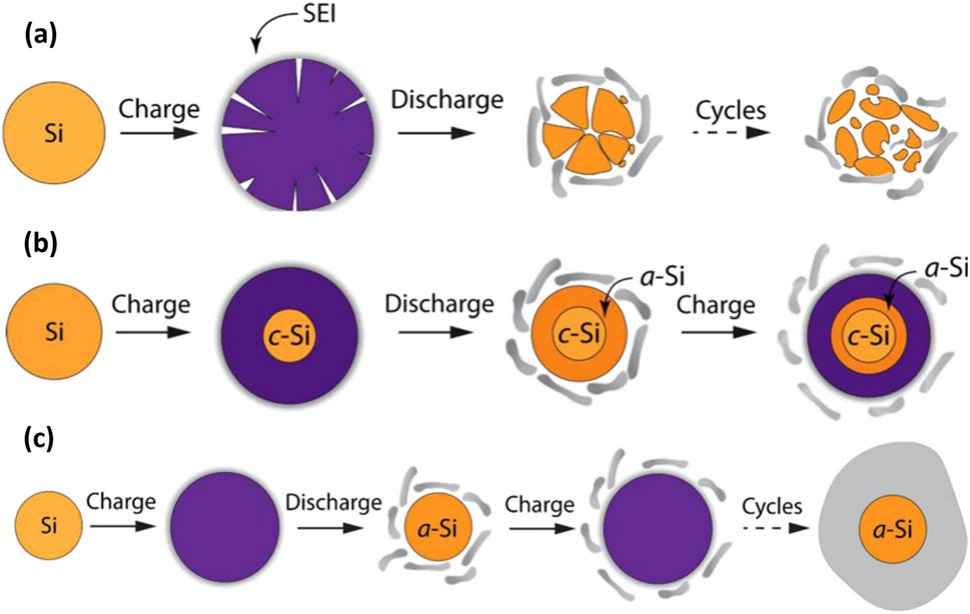
Degradation Mechanisms of Si Anode—**a** Demonstrates the fracture and pulverization of Si electrode materials as a key challenge, **b** addresses lithiation retardation due to compressive stress, which reduces rate performance and lowers the effective capacity, and **c** shows how large volume changes during operation can induce unstable SEI growth on the electrode surface. Each panel offers a focused examination of these mechanisms, presenting critical insights into the durability and performance of Si-based anode materials. Reproduced with permission from Ref. [26]. Copyright 2017, Springer Nature

Several groups [30, 31] delved into the failure mechanism to elucidate the reasons behind the Si anodes’ diminished cycling performance. Their findings can be summarized as:

1. The rapid capacity decay chiefly arises from the considerable volume change (~ 400%) during lithiation and delithiation. Consequently, the contact area between the conductive region and Si is reduced, elevating resistance. These significant volume changes cause pulverization, resulting in the material detaching from the current collector, leading to poor ion and electron transport.
2. A SEI forms during the discharging process, largely attributed to electrolyte decomposition on the anode surface at diminished voltages. Since the particles contract during delithiation, the SEI layer fractures, exposing fresh Si to the electrolyte and compromising electrochemical performance. Figure 2 represents various processes leading to the degradation of the Si electrode throughout the charging and discharging cycles. This degradation can be attributed to a series of mechanisms:

- *Fracture and Pulverization* (Fig. 2a) Lithiation-induced volumetric expansion places significant stress on the Si electrode, leading to its chemomechanical fracture. Such fractures disrupt electrical connectivity between the active materials, current collectors, and electrolytes. This disconnection culminates in swift capacity fading and compromises the cyclability of the battery.
- *Lithiation Retardation* (Fig. 2b) The expansion during lithiation also induces a large compressive stress inside the active materials. This stress acts as a barrier to further lithiation, causing what is referred to as “lithiation retardation”. In severe instances, this compressive stress can completely halt lithiation. The inner core of the active materials, under these conditions, remains untouched by the electrolyte, thus diminishing the effective capacity of the electrode.
- *Unstable SEI Growth* (Fig. 2c) Repeated lithiation/delithiation cycles, coupled with significant volumetric changes, cause the SEI film on the active materials to constantly break and reform. This unstable growth of the SEI leads to the transformation of cyclable lithium ions in the electrode and electrolyte into inert lithium compounds within the SEI. Over time, this phenomenon can result in lithium exhaustion, rendering the battery ineffective.

Building on these observations, recent studies have shed light on additional dimensions of Si anode failure mechanisms [32]. Key insights include the critical role of the SEI layer’s composition and dynamics in electrode longevity. The nature of the SEI, influenced by electrolyte type and operational conditions, significantly impacts the mechanical and chemical stability of Si anodes. Moreover, microstructural changes in Si, such as phase transformations and crystallinity variations during cycling, have emerged as vital factors contributing to capacity fading. These advanced understandings are instrumental in developing effective strategies for improving the resilience and efficiency of Si-based anodes in LIBs.

## Strategies to Improve the Electrochemical Performance of Si-based Anodes

Various strategies have been employed to enhance the electrochemical performance of Si-based anodes, with the primary aim of improving cyclic stability and electrode capacity. An effective approach involves the use of Si/C composites, where Si acts as the high-capacity active material, and the carbon matrix crucially buffers the volume expansion, enhances electrical conductivity, and stabilizes the SEI layer [33–37]. Moreover, the fabrication of nanostructures has become a prevalent method for boosting the performance of Si-based anode materials. Nanostructured Si materials are classified into four categories based on their dimensions: 0D, 1D, 2D, and 3D. The unique attributes of these nanomaterials play a significant role in dictating the electrochemical behavior of the anode materials. For instance, ion and electron transport pathways differ among nanomaterials of varying dimensions. Furthermore, the volume changes that Si-based anode materials undergo during cycling are influenced by the size and morphology of the nanostructures. These methodologies offer significant potential in elevating the performance of Si-based anodes, thereby facilitating the evolution of high-performance LIBs.

### Carbon-Si Composites

In this section, we delve into the realm of Carbon-Si composites, a key area in the advancement of silicon-based anodes for LIBs. This segment focuses on the synergistic integration of Si with various forms of carbon in 1D, 2D, and 3D structures. We discuss the role of carbon materials such as nanofibers, nanotubes, and graphene in constructing effective conductive networks and robust matrices for Si-based anodes. It’s important to note that while 0D structures are equally crucial in the context of Si-Carbon composites, their unique properties and applications warrant a separate discussion, which is addressed in detail in the 'Nanostructured Si Anode Materials' section. Here, we aim to elucidate the role of higher-dimensional carbon structures in enhancing electrical conductivity, mechanical stability, and thermal endurance of silicon-based anodes, particularly addressing challenges like volume changes during charge/discharge cycles.

#### 1D Carbon-Si Composites

Carbon nanofibers (CNFs) and carbon nanotubes (CNTs) are prominent types of 1D carbon materials that have garnered significant attention for their use in LIB applications. The composition of Si with either CNTs or CNFs brings several advantages to the table, notably increased electrical conductivity, as well as exceptional mechanical and thermal stability [38]. As such, CNFs and CNTs have proven more effective in constructing conductive networks and providing a superior host matrix for Si-based anode materials that face volume changes during the charging and discharging process.

**Fig. 3:**
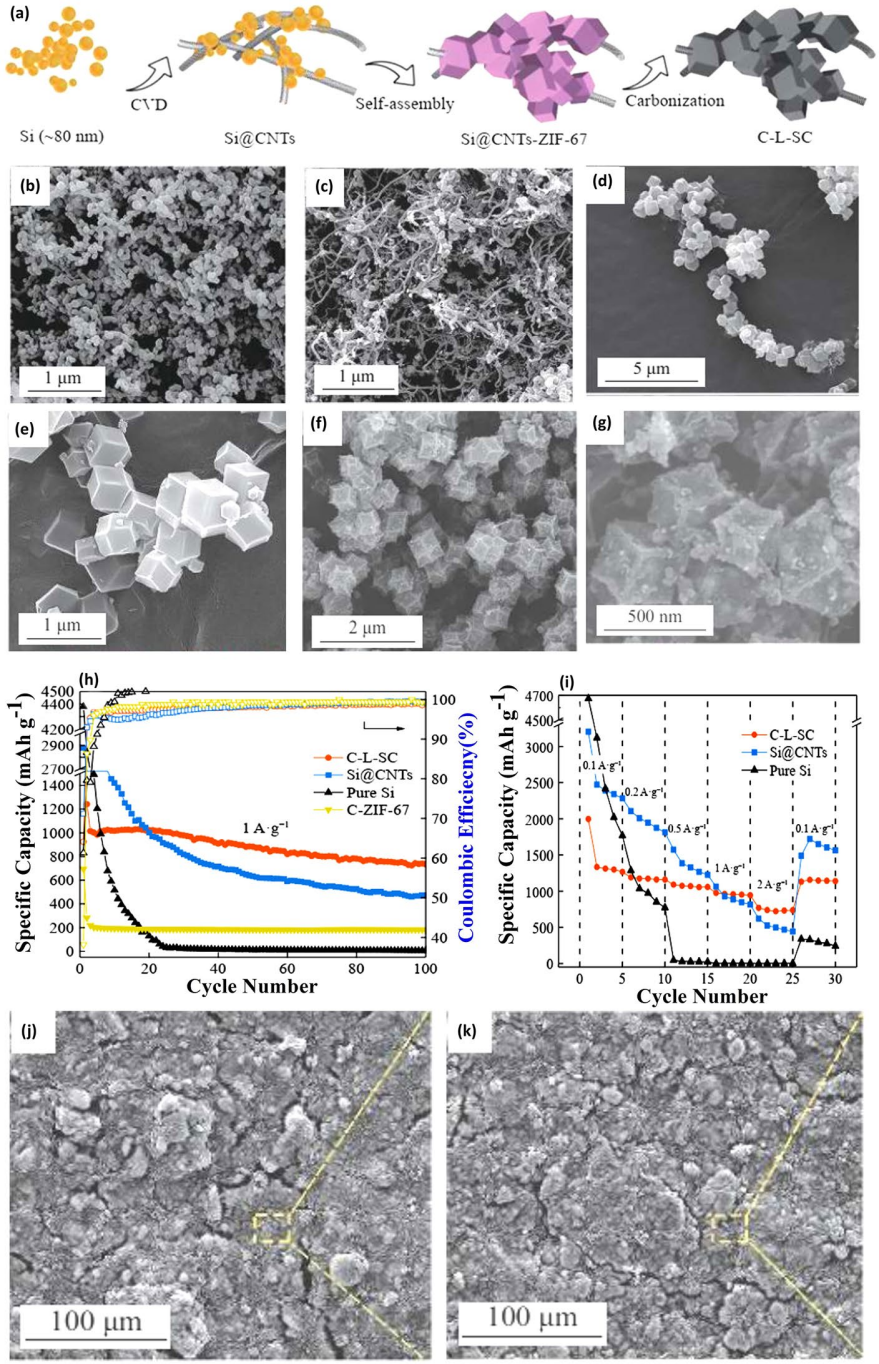
Chain-like Si CNTs (C-L-SC) Preparation and Evaluation—**a** Schematic illustration of the preparation process for chain-like Si CNTs (C-L-SC). SEM images showcasing **b** the morphology of pure Si, **c** Si encapsulated in carbon nanotubes (Si@CNTs), **d**, **e** Si@CNTs after ZIF-67 incorporation (Si@CNTs-ZIF-67), and **f**, **g** the final chain-like Si CNTs structure (C-L-SC). **h** Compares the cycling performances of pure Si, Si@CNTs, C-ZIF-67, and C-L-SC electrodes, demonstrating the enhanced durability and capacity retention of C-L-SC. **i** Rate capability tests for pure Si, Si@CNTs, and C-L-SC electrodes, highlighting the improved performance of C-L-SC under various current densities. SEM images of C-L-SC electrode **j** before and **k** after 100 cycles at 0.5 A g^−1^, illustrating the structural integrity and stability of C-L-SC over extended cycling. Reproduced with permission from Ref. [42]. Copyright 2021, Springer Nature

Before delving into specific studies, it's crucial to acknowledge the significance of ICE in the performance evaluation of silicon-based anodes. Nanosilicon, often used in conjunction with carbon materials like CNTs, can induce more side reactions and form thicker SEI due to its high specific surface area and energy, potentially affecting the ICE. Hence, alongside capacity metrics, the ICE is a vital parameter to assess the initial energy efficiency of these composites. Wang et al. [39], in a pioneering effort, used chemical vapor deposition (CVD) to grow CNTs on both crystalline and amorphous Si. The structure that resulted from this technique offers the rapid transportation of ions and electrons, achieving a high CE and a notable reversible capacity of 2000 mAh g^−1^. Significantly, the ICE of this composite was reported at 80.3%, highlighting its efficiency in the first cycle and indicating a balance between capacity and initial energy retention. Here, the incorporation of CNTs by Wang et al. demonstrates the essential role of 1D carbon structures in enhancing the electrical conductivity and mechanical stability of Si. The CNTs form a conductive network that efficiently manages electron transport and accommodates the volumetric expansion of Si during lithiation, crucial for the observed high CE and capacity. Similarly, Zhang et al. [40] innovated a three-dimensional (3D) Si/CNT composite, ensuring that Si nanoparticles are effectively interconnected electrically via CNTs. This composite, when utilized as an anode for LIBs, showcased a capacity of 943 mAh g^−1^ post 100 cycles. Notably, the ICE for this composite was reported at 56%, underscoring the impact of its design on initial energy efficiency. Such data are crucial for understanding the balance between capacity retention and initial energy utilization in nanosilicon composites. Taking another innovative approach, Su et al. [41] crafted a 3D porous material constituted of SiO_2_ and Si that was seamlessly decorated with CNTs in situ, termed as pSS/CNT. This unique composite offers superior stability during cycling and boasts an improved CE. Importantly, the ICE of the pSS/CNT composite is reported to be 70%. This figure is crucial as it provides a measure of the initial energy efficiency of the composite, highlighting its effectiveness in the first cycle and contributing to a comprehensive understanding of its overall electrochemical performance. Peng et al. [42] ventured to design a chain-like Si CNT architecture (C-L-SC), integrating ZIF-67 derived porous carbon encapsulating Si nanoparticle with CNTs acting as connectors between the carbon shells. The manufacturing procedure of C-L-SC involves the growth of CNTs upon SiNPs via CVD to generate a tangled cluster structure first, and the growth of ZIF-67 on the surface, followed by carbonization (Fig. 3a). Figure 3b showcases the morphology of pure Si NPs, which exhibit a mean diameter of ~ 80 nm and a relatively uniform distribution. After CVD process, the CNTs uniformly grew on the nanosized Si, intertwining among themselves as seen in Fig. 3c. Figure 3d, e illustrates ZIF-67 crystals growing along the Si@CNTs, in which a chain-like structure formed. Upon carbonization, the C-L-SC retained its original structure, with ZIF-67 crystals maintaining their dodecahedral morphology as depicted in Fig. 3f, g. Figure 3h, i provides a comparative analysis of the cycling performances and rate capability of various compositions, including pure Si, Si@CNTs, C-ZIF-67, and C-L-SC electrodes. Impressively, the C-L-SC composite exhibited significant rate capacity and cycling stability, maintaining a capacity of 732 mAh g^−1^ at 2 A g^−1^ and retaining 72.3% of this capacity over 100 cycles at 1 A g^−1^. To assess the anode material's stability, SEM imaging captured the morphological evolution of the C-L-SC electrode before (Fig. 3j) and after cycling (Fig. 3k). Remarkably, even after 100 cycles, the electrode maintained a complete and unaltered structure, devoid of new surface cracks. This series of studies further emphasizes the significance of 1D carbon structures. The mechanical stability provided by CNTs and CNFs is key in alleviating the strain associated with Si’s volume changes during battery operation. This contributes to improved cycle stability and capacity retention, as evidenced in these Si/C composites.

In a similar realm of innovation, Deng et al. [43] conceived a structure by embedding Si nanoparticles within the core of double-layered CNTs, resulting in a sandwich-like configuration. This configuration significantly boosted the electrochemical attributes of Si electrodes. In a noteworthy methodological shift, CNT arrays were synthesized using liquid paraffin, replacing traditional unsaturated hydrocarbons via the CVD method. This unique design ensured that the resultant material exhibited stellar rate performance and excellent cycling stability, retaining capacities of 1310 mAh g^−1^ at 0.1 A g^−1^ post 100 cycles and 1050 mAh g^−1^ at 1 A g^−1^ post 500 cycles, alongside a 98% CE. In another commendable effort, Zhang et al. [44] synthesized Si/Cu_3_Si@C composites encased within a mesh of CNTs (SCC-CNTs) by amalgamating ball milling and CVD techniques. Comprising of conductive Cu_3_Si, an amorphous carbon layer, interconnected CNTs, and etched pores, these elements collaboratively enhance electronic conductivity and Li^+^ diffusion. The electrochemical processes involved also counteract the volume expansion of the Si anode. When juxtaposed against pure Si, SCC-CNTs composites manifest considerably superior electrochemical performance, recording a discharge capacity of 2171 mAh g^−1^ at 0.4 A g^−1^, an ICE of 85.2%, and a retained capacity of 1197 mAh g^−1^ after 150 cycles. In these examples, the 1D carbon matrix notably boosts electron conductivity while providing a mechanical buffer against Si volume expansion during cycling. This synergy between the conductive carbon network and Si nanoparticles is instrumental in achieving enhanced rate performance and cycling stability in the Si/CNT and Si/Cu_3_Si@C composites.

**Fig. 4:**
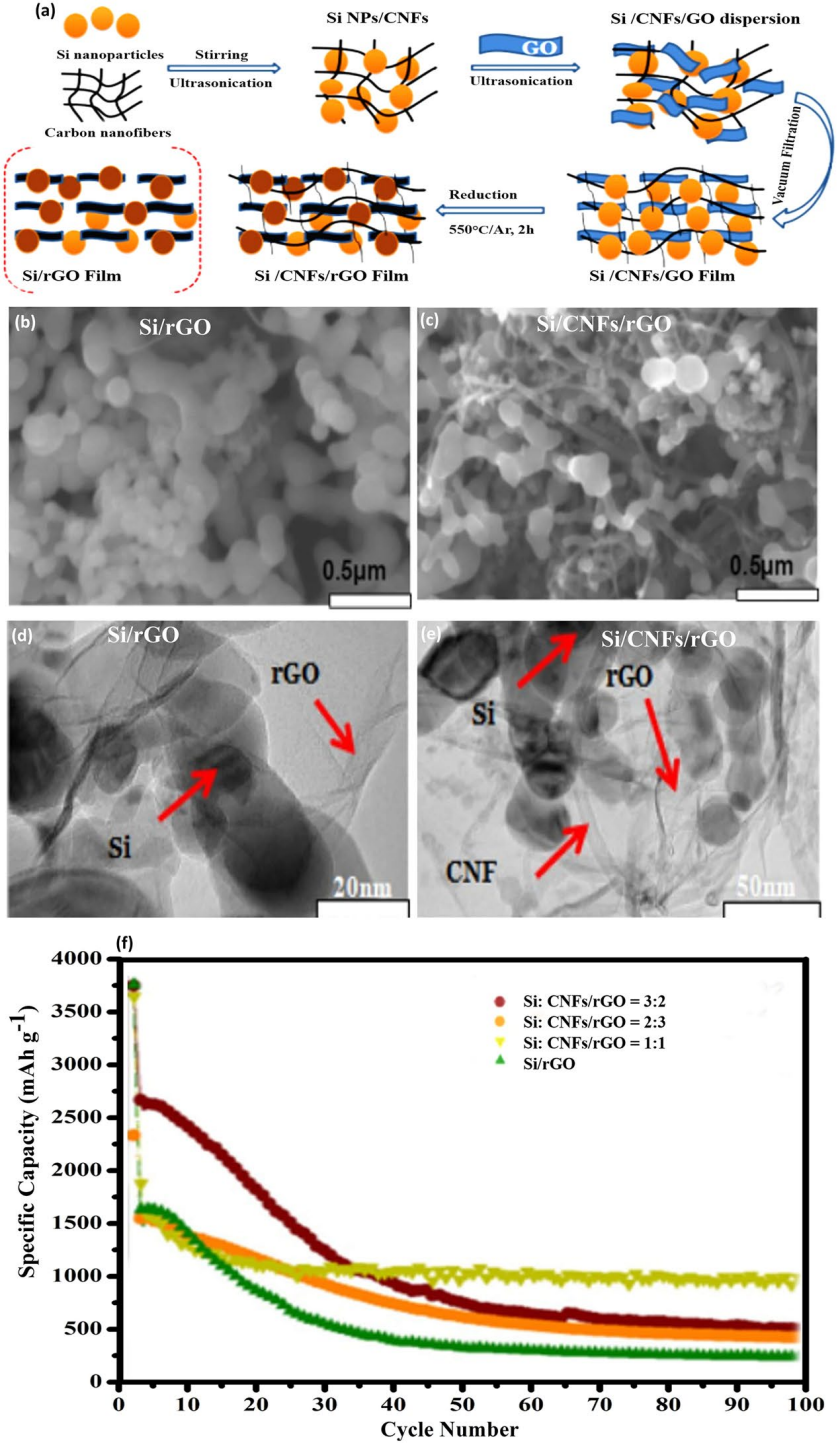
Fabrication and Analysis of Si/CNF/rGO Composite Films—**a** Illustration of the preparation process for Si/CNF/rGO and Si/rGO composite films, outlining the steps taken to synthesize these materials. SEM images of **b**, **c** the Si/rGO and Si:CNF/rGO = 1:1 composites, respectively, showing the morphology and distribution of materials within the composites. TEM images of **d**, **e** similarly showcase the microstructure of Si/rGO and Si:CNF/rGO = 1:1 composites, providing a closer look at the nanoscale interactions and structure. **f** Cycling performances of the synthesized Si/rGO and Si/CNF/rGO composites at ratios of 1:1, 2:3, and 3:2, evaluated at a current density of 0.1 A g^−1^, highlighting their electrochemical stability and capacity retention over cycles. Reproduced with permission from Ref. [46]. Copyright 2021, Springer Nature

CNFs, due to their distinct properties, have been extensively incorporated in LIB applications. Their robustness in terms of chemical and thermal stabilities is complemented by their commendable electrical conductivity. Xu et al. [45] employed a fusion of electrospinning and electrospray techniques to produce 3D Si/C fiber paper, exhibiting a formidable electrochemical performance of 1200 mAh g^−1^ after a span of 100 cycles. In a similar vein, Lee et al. [46] demonstrated the potential of Si/CNF composite films for anode applications. These films, developed through a filtration method followed by a thermal reduction process, showcased a significant enhancement in LIB performance, as depicted in Fig. 4a. The SEM images (Fig. 4b, c) reveal a dense, intertwined architecture where CNFs support Si particles, forming a robust three-dimensional matrix. This configuration is conducive to efficient electron and ion transport and effectively manages the volumetric alterations of Si particles throughout charge/discharge cycles, ensuring exceptional mechanical stability. TEM analyses (Fig. 4d, e) further confirm the dense packing and uniform distribution of Si particles within the CNFs. This structural design is pivotal for achieving superior specific capacity and excellent cycle and rate performances.

In summary, while 1D Si/C composites such as Si/CNTs and Si/CNFs show significant potential in enhancing charge transfer and lithium-ion diffusion for improved electrode cycling stability, they present unique challenges. Their complex fabrication processes require innovative and cost-efficient strategies, and their adaptation to high-performance applications demands careful consideration of mass loadings and economic feasibility. These challenges not only highlight the critical need for ongoing research and development within the realm of 1D structures but also pave the way for investigating the next frontier in carbon-Si composites—the realm of two-dimensional (2D) structures. The transition to 2D carbon-Si composites presents an opportunity to build on the foundations laid by 1D composites while exploring new structural benefits and solutions to persisting challenges.

#### 2D Carbon-Si Composites

2D materials offer distinct advantages as anodes. They can shield electrodes from abrasion, enhance the synergy of Li-ion deposition, assist Li ions in traversing through electrolytes and electrodes, and bolster stability at elevated temperatures. Among these materials, graphene stands out as the predominant 2D carbon substance, boasting a surface area of 2630 m^2^ g^−1^ and exceptional mechanical, electrical, and thermal properties [47, 48]. It’s a favored choice for energy storage, extensively utilized in LIBs, Na-ion batteries, supercapacitors, and Li-S batteries [49]. The significant transformation brought about by 2D materials in Si-based anodes can be attributed to several key mechanisms. The planar structure of materials like graphene provides a large surface area for uniform Li-ion deposition and facilitates rapid electron and ion transport. This not only improves the electrical conductivity within the electrode but also effectively manages mechanical stresses during lithiation/delithiation processes. The synergy between the 2D carbon structure and Si contributes to both the electrochemical efficiency and mechanical resilience of the anode. Such structural benefits are critical in overcoming the challenges of electrochemical inefficiency commonly experienced with Si anodes. Su et al. [50] devised an efficient approach to creating a Si@G composite with a spherical morphology using a simple spray drying method, eliminating the need for post-annealing. This composite, characterized by its core–shell structure, significantly improves both electric and ionic conductivities due to the synergistic interaction between the spherical shape and the multi-layered graphene arrangement. A crucial element of this approach is the formation of a void within the Si@G structure, designed to accommodate Si's inherent volume expansion during battery operation. The resulting robust Si@G composite demonstrates an impressive initial discharge capacity of 2882.3 mAh g^−1^ and an admirable 86.9% ICE at 0.2 A g^−1^.

**Fig. 5:**
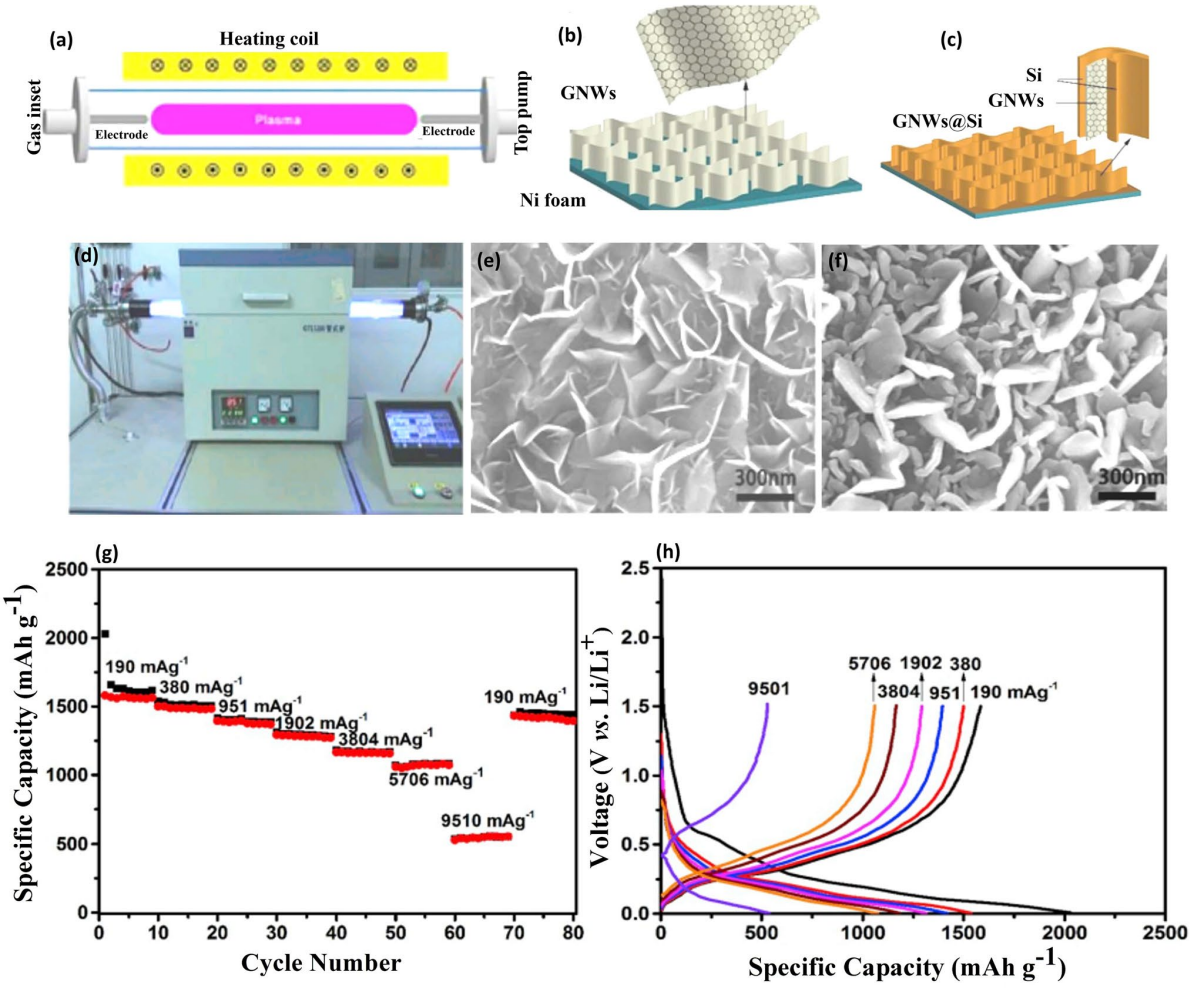
RF Plasma-Enhanced Fabrication of GNWs@Si Composites and Their Electrochemical Evaluation in LIBs—**a** Schematic diagram of the radio-frequency plasma-enhanced horizontal tube furnace deposition system. **b** Schematic images depicting the growth of graphene nanowalls (GNWs) on Ni foam. **c** Schematic representation of GNWs@Si composite preparation on Ni foam. **d** Actual image of the radio-frequency plasma-enhanced horizontal tube furnace deposition system. **e** SEM images showcasing the morphology of GNWs. **f** SEM images of GNWs@Si composite, illustrating the composite’s detailed structure. **g** Voltage profiles of LIBs based on GNWs@Si composite at varying current densities from 190 to 9510 mA g^−1^. **h** Galvanostatic charge–discharge capacity of the same, depicting the performance across different rates. Reproduced with the permission from Ref. [52]. Copyright 2019, Elsevier

In a related study, Jamaluddin et al. [51] presented a novel Si@N-G composite, where Si nanoparticles (SiNPs) serve as the core and electrochemical exfoliation-derived nitrogen-doped graphene (N-ECG) forms the shell. This composite underwent further annealing with H_2_ and NH_3_ to optimize its properties as an anode material. The incorporation of N-ECG, led to significantly improved electrical conductivity and ion mobility. These enhancements were particularly prominent in the Si@N-ECGB microball structure, demonstrating a high initial discharge capacity of 2604.5 mAh g^−1^, and a CE of 85.2%. The superior electrochemical performance can be attributed to its facilitation of an effective lithiation/delithiation process. Lin et al. [52] devised an innovative approach to fabricate Si deposited graphene nanowalls (GNWs) within Ni foam matrix. As illustrated in Fig. 5a–c, GNWs were first grown on Ni foams using a plasma-enhanced tube furnace deposition system. Then Si materials were precisely deposited onto the as-grown GNWs via magnetron sputtering, ensuring a uniform, smooth, and conformal coverage of Si thin film on both sides of the GNWs, as depicted in Fig. 5f. The unique architecture of these 3D GNW networks facilitates enhanced electron transport. Notably, the Si thin film with a thickness of several tens of nanometers, acts as a buffer against the pulverization issue arising from volume expansion. Figure 5g, h showcases remarkable adaptability across a diverse range of current rates when the GNWs@Si composite applied in LIBs. The composite retained a commendable discharge specific capacity of 1450 mAh g^−1^ at 190 mA g^−1^ even after seventy dynamic cycles. Such exemplary performance underscores the potential of the GNWs@Si nanocomposite, attributing its efficiency to the three-dimensional network structure of GNWs, which bolsters the conductivity and minimizes electrode polarization at elevated rates. For instance, the Si@G composite developed by Su et al. demonstrates how the 2D graphene structure, with its high conductivity and mechanical stability, synergistically interacts with Si to enhance the composite's overall performance, particularly in terms of capacity and cycle life. MXenes, another breed of 2D carbon materials, consist of nitrides and carbides with multi-atomic layer thicknesses, finding uses in various battery components [53–56]. Guo et al. [57] introduced the MXene/Si@SiO_*x*_@C composition, which displayed robust cycling performance influenced by Si concentration. At 72.8 wt% of Si, the anode exhibited a stellar capacity of 470 mAh g^−1^ at 10 C across 1000 cycles, credited to the MXene layers' high electronic conductivity, modifiable layer spacing, and abundant covalent bonding.

In summary, 2D materials have significantly transformed the landscape of anode enhancements, particularly with their incorporation into Si-based anodes. Their unique structural properties, combined with impressive electrical conductivity, provide a tactical advantage in overcoming the common setbacks experienced with Si anodes, notably regarding electrochemical inefficiency. By enhancing electron conductivity within electrodes, these 2D materials, including graphene and other layered materials, have demonstrated substantial advancements in battery performance metrics. These enhancements are evident in their impressive specific and volumetric capacities, along with and their ability to withstand substantial mechanical stresses encountered during lithiation/delithiation process.

However, although 2D materials represent a revolutionary stride forward, they constitute just one facet of the broader exploration into spatially complex carbon-Si composites. Progressing from 2 to 3D frameworks ushers in a new era of possibilities in energy storage technology. The subsequent section will shift our discussion to 3D carbon-Si composites, where we will investigate how these intricate structures continue to push the boundaries of electrochemical performance. As we delve into 3D configurations, we anticipate unveiling innovative strategies to navigate the inherent challenges of Si-based anodes, potentially leading to even more robust, efficient, and durable LIBs.

#### 3D Carbon-Si Composites

Porous carbon and graphite stand out as the two primary categories of 3D carbon materials extensively utilized in LIBs. The widespread use of porous carbon as a support material in LIBs is attributed to its ability to facilitate rapid ion and electron mobility, coupled with its generous void space. A groundbreaking study led by Li’s group [58] detailed the fabrication process of gigaporous carbon microspheres. This process involved emulsifying poly (styrene-co-vinylbenzyl chloride), oleic acid, and Si powder, resulting in Si-embedded porous microspheres that was subsequently encapsulated in situ with poly (urea–formaldehyde) (PUF). By incorporating an extra carbon source and adjusting the pH to accomplish the carbon encapsulation, carbonizing the resulting material was carbonized, giving rise to the final carbon-encapsulated porous Si/C microsphere. Prior to Si implantation, these microspheres exhibited a profusion of micropores and were infused with increasing quantities of micron-sized Si powder. They meticulously analyzed both micron-sized (u-Si) and nanosized (n-Si) Si particles. The improved retention of n-Si over u-Si is attributed to its reduced volume fluctuation. Furthermore, the extra carbon layer on the Si/C microsphere fortified the structural integrity of the electrode. The carbon-encapsulated n-Si/C microsphere debuted with an initial capacity of 3320 mAh g^−1^ and remarkably retained 90% of this capacity over after 100 cycles, benefiting from the leveraging the robust porous carbon framework.

**Fig. 6:**
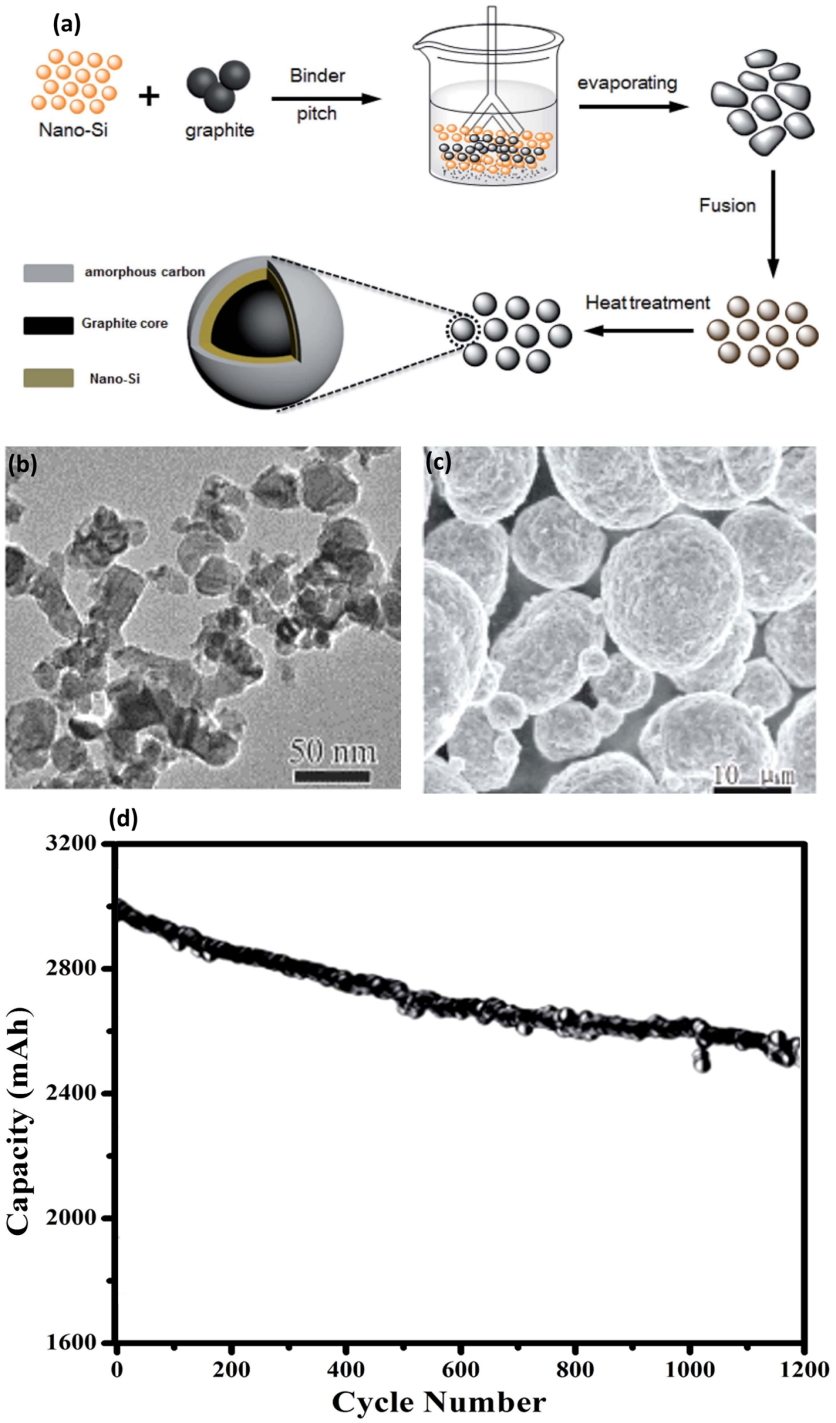
Preparation and Electrochemical Performance of Si–G/C Composite for High-Energy–Density Batteries—**a** Schematic preparation procedure of the Si–G/C composite, illustrating the step-by-step synthesis process. **b** Typical TEM profiles of nano-Si, providing insights into the nanostructure of the silicon used in the composite. **c** SEM images of the Si–G/C composite, highlighting the microstructural integration of silicon with graphene/carbon. **d** Cycling performance of the Si–G/C composite in prototype full-cell high-energy–density batteries, demonstrating stability and capacity retention over 1200 cycles at a charge/discharge rate of 0.5 C. Reproduced with the permission from Ref. [59]. Copyright 2018, Royal Society of Chemistry

Si/graphite composites are particularly captivating because graphite augments the composite's capacity while serving as a Si matrix, dispersing volume expansion and facilitating electrical connection. Xiao et al. [59] pioneered in the realm of electrode material design with their innovative Si/graphite/carbon (Si-G/C) composite. As schemed in Fig. 6a, a Si/graphite precursor was first fabricated by thorough mixing of nano-Si particles, graphite, pitch and polyvinyl pyrrolidone (PVP), which was then underwent a spherification process in a fusion machine, followed by a heat treatment at 900 °C, resulting in the Si-G/C composite. Figure 6b exemplifies the TEM depictions of the Si nanoparticles with 30–50 nm in diameter, exhibiting tendencies of aggregation owing to their elevated surface energies. A comprehensive structural profile of the Si-G/C composite is presented in Fig. 6c. Figure 6d indicates that the full cell, integrating the Si-G/C composite anode with the NCA cathode, demonstrates an initial cell capacity of 3000 mAh and maintains an impressive 81% of this capacity over 1200 cycles. This underscores the potential of the Si-G/C composite as a robust and reliable component in advanced battery assemblies.

Elsewhere, Li et al. [60] developed a porous Si/C composite, which was then integrated into a graphite-blended anode. This composite consistently achieved a capacity of 550 mAh g^−1^ across 200 cycles. Wu et al. [61] innovatively harnessed pinecones to craft an environmentally benign porous carbon (PPC) envisioned as an ideal matrix for Si/carbon composite anodes. Leveraging the intrinsic porous architecture of pinecones, they proficiently encased Si nanoparticles within the PPC substrate, culminating in the PPC/Si composite's birth. This composite, bolstered by the electro-conductive PPC network and minuscule Si nanoparticles, enhances both electron and ion movements. Its porous attributes not only guarantee optimal electrolyte permeation but also make room for Si’s volume expansion during cycling. Consequently, the PPC/Si composite exhibits a stellar rate performance-478.4 mAh g^−1^ at 2 A g^−1^ and remarkable cycling endurance-720.6 mAh g^−1^ at 0.2 A g^−1^ after 300 cycles.

To encapsulate, 3D carbon coatings, when applied to Si surfaces, are meticulously engineered to shield Si particles, preventing their direct exposure to electrolytes. Instead of interacting with Si directly during electrochemical processes, the electrolyte constructs a stable SEI film in tandem with the carbon layer. This robust carbon layer absorbs the volume expansion of Si, ensuring the prevention of Si disintegration. The unique yolk-shell structure of Si/3D carbon composites creates ample space for Si particle fluctuations, negating their detachment of the particle from the electrode, thereby preserving their electrochemical vitality. 3D carbon coatings have undeniably demonstrated their efficacy in shielding Si anodes and sustaining exceptional battery performance. However, it’s important to note that producing 3D carbon-coated Si tends to be costlier, and refining the process demands meticulous attention and precisions.

In summary, 3D carbon-Si composites represent a pinnacle in anode engineering, harnessing the structural benefits of three-dimensional carbon materials to address fundamental issues associated with Si anodes. These composites excel in accommodating Si’s volume expansion, enhancing electrical connectivity, and ensuring structural integrity during rigorous cycling. Innovations, particularly in the synthesis of gigaporous carbon microspheres and unique Si/graphite/carbon composites, have underscored the feasibility and performance superiority of these 3D constructs. While challenges in cost and fabrication complexity underscore the need for further optimization, the advancements within this domain lay a solid foundation for the next phase of anode material research. Table 3 provides the cycle stability of various Si/C-based for LIBs.

**Table 3:** Cycle stabilities of various Si/C- based anodes for LIBs

Si Anodes	Current density (A g^−1^)	Cycle number	Remaining capacity (mAh g^−1^)	Refs.
Si/C	0.3	100	611.3	[62]
Si/p-C(N-SPC)	0.4	100	1607	[63]
Si@viod@C	–	40	500	[64]
Si@C@viod@C	0.1	50	~ 1350	[65]
Porous Si/C	0.5	30	759	[66]
Meso-Si/C	1	1000	990	[67]
Porous Si/C nanotubes	0.2	200	1300	[68]
Si/graphite/C	0.5	300	~ 400	[69]
Si@C@CNTs&CNFs	0.3	50	1195	[70]
Si/CNTs	0.42	100	1000	[39]
Si/CNTs	42	100	800	[71]
Si@HC/CNFs	0.2	100	~ 1020	[72]
Si/graphene	0.1	200	1500	[33]
Si/rGO	0.1	100	1433	[73]
Si/C/graphene	0.2	100	760	[74]
Si@C-rGO	0.3	400	931	[75]
M-pSi@C	1	250	1702	[76]
Si/C	0.1	100	941	[77]
Si/C	0.5	100	605.43	[78]
Si/C	2	400	1283	[79]
Si/C/rGO	1	270	1004	[80]
C-Si@graphite	4.2	1000	~ 900	[81]
C@void/Si-G	8.4	200	1082.7	[82]
Si/Cu/Cu_3_Si@C	2.1	500	984	[83]

In 3D carbon-Si composites, the structural benefits of three-dimensional carbon materials play a crucial role in enhancing the performance of Si anodes. The porous nature of materials like gigaporous carbon microspheres and graphite provides extensive void space, facilitating rapid ion and electron mobility. This architecture is instrumental in managing the volume expansion of Si during cycling, a key challenge in Si anode utilization. Additionally, the 3D carbon framework ensures structural integrity and consistent electrical connectivity, further contributing to the robustness and efficiency of the composite. These structural advantages, combined with the unique yolk-shell configurations, offer an effective buffer against mechanical stresses and electrolyte interactions, thereby significantly enhancing the durability and electrochemical performance of Si anodes.

As we transition into new strategic explorations, the upcoming sections will focus on the burgeoning field of nanostructured-based Si anode materials. The shift toward nanotechnology signifies our continual pursuit of refining performance and longevity in LIBs, where even finer control at the molecular level opens new horizons for material capabilities and electrochemical outcomes.

### Nanostructured Si Anode Materials

This section is dedicated to exploring nanostructured Si anode materials in LIBs, including a special focus on zero-dimensional (0D) Si-based materials. While our discussion in ‘Carbon-Si composites’ primarily addresses 1D, 2D, and 3D structures due to their distinct characteristics and integration methods with carbon materials, in this section, we highlight the unique properties and significance of 0D Si structures, such as Si nanoparticles, and their composites with carbon materials. These nanostructures are pivotal in addressing key challenges associated with Si anodes, like volume expansion and electrical conductivity. Through this section, we aim to provide a comprehensive overview of the advancements and techniques in nanostructuring Si, particularly emphasizing the role of 0D structures and their composites, in enhancing the efficiency and durability of LIBs.

#### 0D Si-Based Anode Materials

Si nanoparticles, classified as zero-dimensional Si materials, have become highly promising candidates for LIBs for two primary reasons:

1. Their diminutive size is essential for alleviating stress and preventing Si damage during the lithiation/delithiation processes, enhancing cycling performance [84, 85].
2. Their synthesis techniques are well-established, making them commercially accessible [86].

**Fig. 7:**
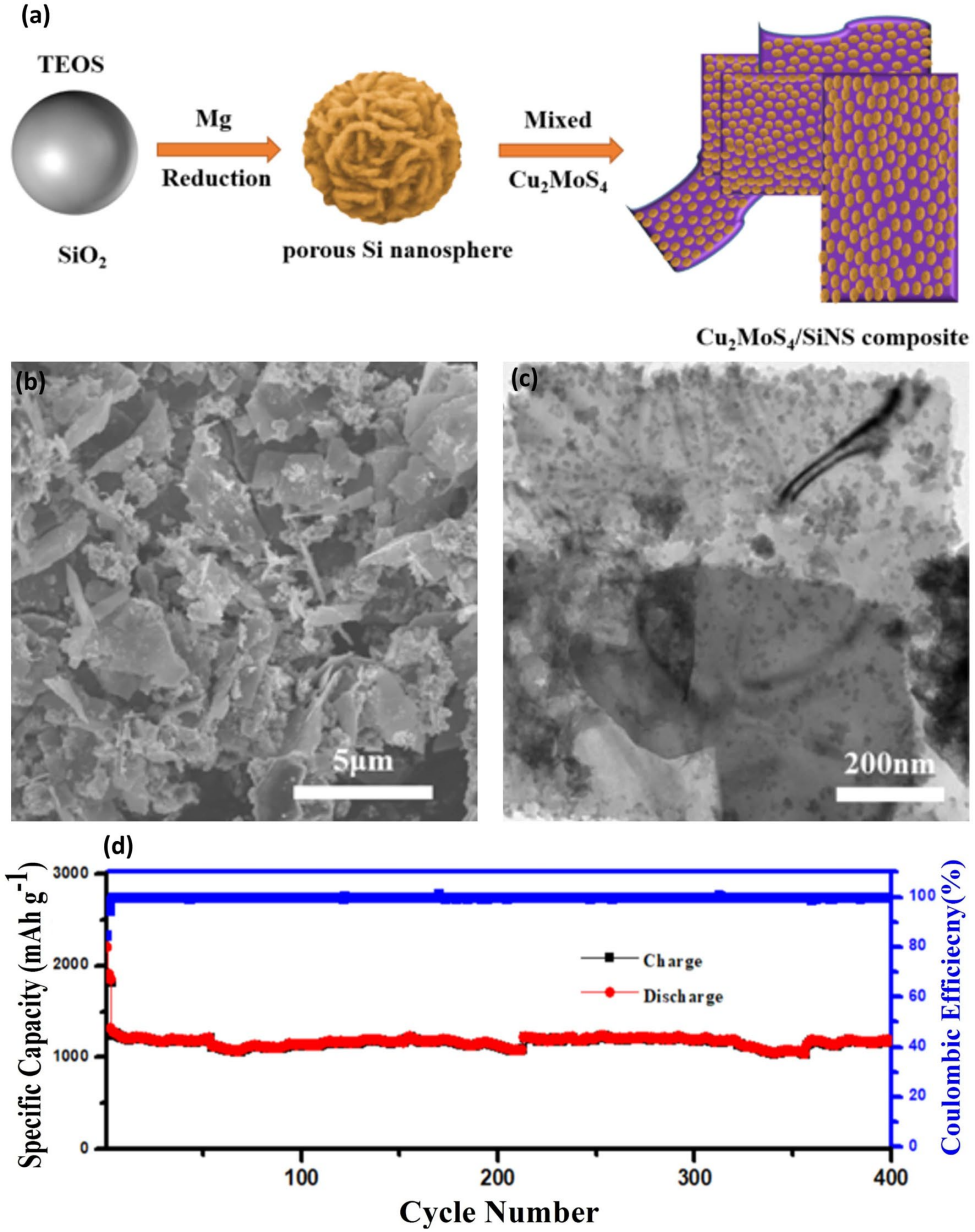
Synthesis and Electrochemical Evaluation of Cu_2_MoS_4_/SiNS Composite for Anode Material—**a** Illustrates the synthetic strategy process for creating Cu_2_MoS_4_/SiNS through the self-assembly of composite porous Si nanospheres with Cu_2_MoS_4_, detailing the steps involved in the composite formation. **b** SEM image showing the morphology of the self-assembled Cu_2_MoS_4_/SiNS anode material, highlighting the microstructural characteristics. **c** TEM images of the Cu_2_MoS_4_/SiNS composite, providing a closer look at the nanostructure of the composite material. **d** Cycling performance of Cu_2_MoS_4_/SiNS demonstrated over 400 cycles at a current density of 2.0 A g^−1^, showcasing the material's durability and electrochemical stability. Reproduced with permission from Ref. [90]. Copyright 2021, American Chemical Society

Ge and colleagues [87] crafted porous Si nanoparticles using through electroless etching and boron doping. Upon incorporating with graphene, the resulting material exhibited a commendable capacity of 1000 mAh g^−1^ after 200 cycles. Wang’s team [88] introduced a mesoporous Si/C composite, synthesized using a self-assembly method induced by evaporating a triblock copolymer in a resorcinol–formaldehyde (RF) resin. This composite featured 100 nm Si nanoparticles evenly dispersed within a mesoporous carbon matrix, boasting a capacity of 1018 mAh g^−1^ over 100 cycles. Epur et al. [89] innovated a binder-free Si/MWCNT anode for LIBs, demonstrating an initial discharge capacity of 3112 mAh g^−1^ at 300 mA g^−1^. Remarkably, it retained 76% of this capacity after 50 cycles, attributed to the robust CNT/Si connection, which ensured minimal volume variation and robust electrical conductivity. Liu et al. [90] introduced an innovative and effective synthesis strategy to produce composite Cu_2_MoS_4_/SiNS materials, achieved through the self-assembly of silicon nanospheres and a two-dimensional Cu_2_MoS_4_ material (Fig. 7a). The resulting Cu_2_MoS_4_/SiNS composite exhibited clear adherence of silicon nanospheres to small particles on the surface of the sheet material (Fig. 7b). TEM analysis further revealed the distinctive sheet structure of the Cu_2_MoS_4_/SiNS composite, showcasing the efficient dispersion of porous silicon within the two-dimensional layered structure (Fig. 7c). This dispersion plays a crucial role in releasing volume expansion and alleviating mechanical stress, providing additional active sites and fast channels for Li^+^ transmission, ultimately enhancing the material’s conductivity. When employed as the anode in LIBs, the Cu_2_MoS_4_/SiNS material displayed significantly improved electrochemical performance. Pushing the boundaries, the material was subjected to fast charging at an increased current density of 2.0 A g^−1^. Impressively, it demonstrated a specific capacity of 1180 mAh g^−1^ with 69.2% capacity retention even after 400 cycles (Fig. 7d). The composite material's remarkable stability and enhanced capacity underscore its potential for advancing the construction of high-performance LIBs.

Jung et al. [91] employed a one-pot hydrothermal technique to produce a SiC composite material, incorporating etching-modified Si nanoparticles and sucrose as a carbon precursor. The proposed SiC composite, which is meso-macroporous and houses a significant quantity of Si nanoparticles (40 wt%) within a 3 μm diameter carbon sphere, presented a high initial capacity of 1300 mAh g^−1^, with an impressive 90% retention after 200 cycles and rapid charge/discharge abilities of just 12 min. Zhou and colleagues [92] developed SiNP@G, achieving a capacity of 1205 mAh g^−1^ over 150 cycles. Liu’s team [93] utilized CVD to design a yolk-shell carbon structure on Si nanoparticles, facilitating Si expansion without rupture during charging and discharging processes.

Si nanoparticles, characterized by their porous and hollow configurations, yield superior electrochemical performance due to several factors:

1. Voids and pores accommodate volume expansion, effectively safeguarding against the volume alterations during charge and discharge processes. This unique feature ensures the structural integrity of the material, allowing it to endure repeated cycles without significant degradation.
2. The porous architecture truncates electron and lithium pathways, minimizing polarization and amplifying rate efficiency [94]. By reducing the distances lithium ions and electrons need to travel, the material can charge and discharge rapidly, making it ideal for high-performance applications.
3. Such structures decrease local current density, leading to reduced stress gradients near particle surfaces and thus enhanced electrochemical performance. By distributing the electrochemical reactions more evenly throughout the material, these nanostructures can sustain their performance over numerous cycles.

Fang et al. [95] unveiled a Si@TiO_2_ core–shell structure, encapsulating Si nanoparticles within a TiO_2_ void. This design demonstrated an impressive capacity of 804 mAh g^−1^ at 0.1 C over 100 cycles, highlighting the potential of core–shell structures in enhancing Si-based electrode materials. Furthermore, through mechanical mixing, Park and associates [96] produced Si/Ti_2_O_3_/rGO, forming ternary nanocomposite with a capacity of 950 mAh g^−1^ after 100 cycles at 100 mA g^−1^. This versatile approach demonstrates the continuous efforts to innovate Si nanostructures, addressing mechanical challenges associated with lithiation and delithiation processes. Their smaller scale not only facilitates rapid Li transfer but also effectively mitigates stress, ultimately leading to improved overall performance.

In the realm of 0D Si-Based Anode Materials, the diminutive size of Si nanoparticles plays a pivotal role. Their nanoscopic scale fundamentally changes the kinetics of lithium insertion and extraction processes. This smaller scale allows for rapid Li-ion transfer and effectively mitigates mechanical stress during the lithiation/delithiation cycles. Additionally, the porous and hollow configurations of these nanoparticles provide voids that accommodate volume expansion, safeguarding the structural integrity during charge and discharge processes. This feature is crucial in preventing significant material degradation over numerous cycles. Furthermore, their synthesis techniques, such as electroless etching and self-assembly, enable the production of Si nanostructures with features like mesoporosity and carbon coating, which further enhance their electrochemical characteristics.

In conclusion, zero-dimensional Si nanoparticles emerge as a revolutionary stride in the realm of LIB anode materials. The strength of these materials lies in their nanoscopic scale, which fundamentally alters the kinetics and mechanics of lithium insertion and extraction processes. Through various innovative approaches, researchers have successfully synthesized Si nanostructures with enhanced electrochemical characteristics. Techniques such as electroless etching, self-assembly, and chemical vapor deposition have led to the development of Si nanostructures with integral features such as mesoporosity, carbon coating, and yolk-shell architectures. These structural refinements address the critical challenges associated with Si’s volume expansion and mechanical degradation during battery operation.

Particularly notable are the strategies involving the incorporation of conductive additives, protective coatings, and structurally reinforcing elements that contribute to prolonged cycle life and improved capacity retention. Examples, including Si/CDs, Si@TiO_2_, and Si/Ti_2_O_3_/rGO composites, have underscored the versatility and adaptability of Si nanoparticles in various composite configurations. Moreover, the deliberate design of voids and pores in these particles serves multiple purposes, from accommodating physical expansion to enhancing ion transport and reducing polarization effects.

As we advance to explore one-dimensional (1D) Si-based materials, the insights gained from 0D Si nanoparticle research set a compelling precedent. The quest continues to optimize Si anodes’ resilience and efficiency, with nanoscale engineering remaining at the forefront of these innovative efforts. The ensuing section will delve into the prospects offered by 1D Si structures, reflecting on their unique geometries and the implications thereof in the continuing evolution of LIB technology.

#### 1D Si-Based Anode Materials

**Fig. 8:**
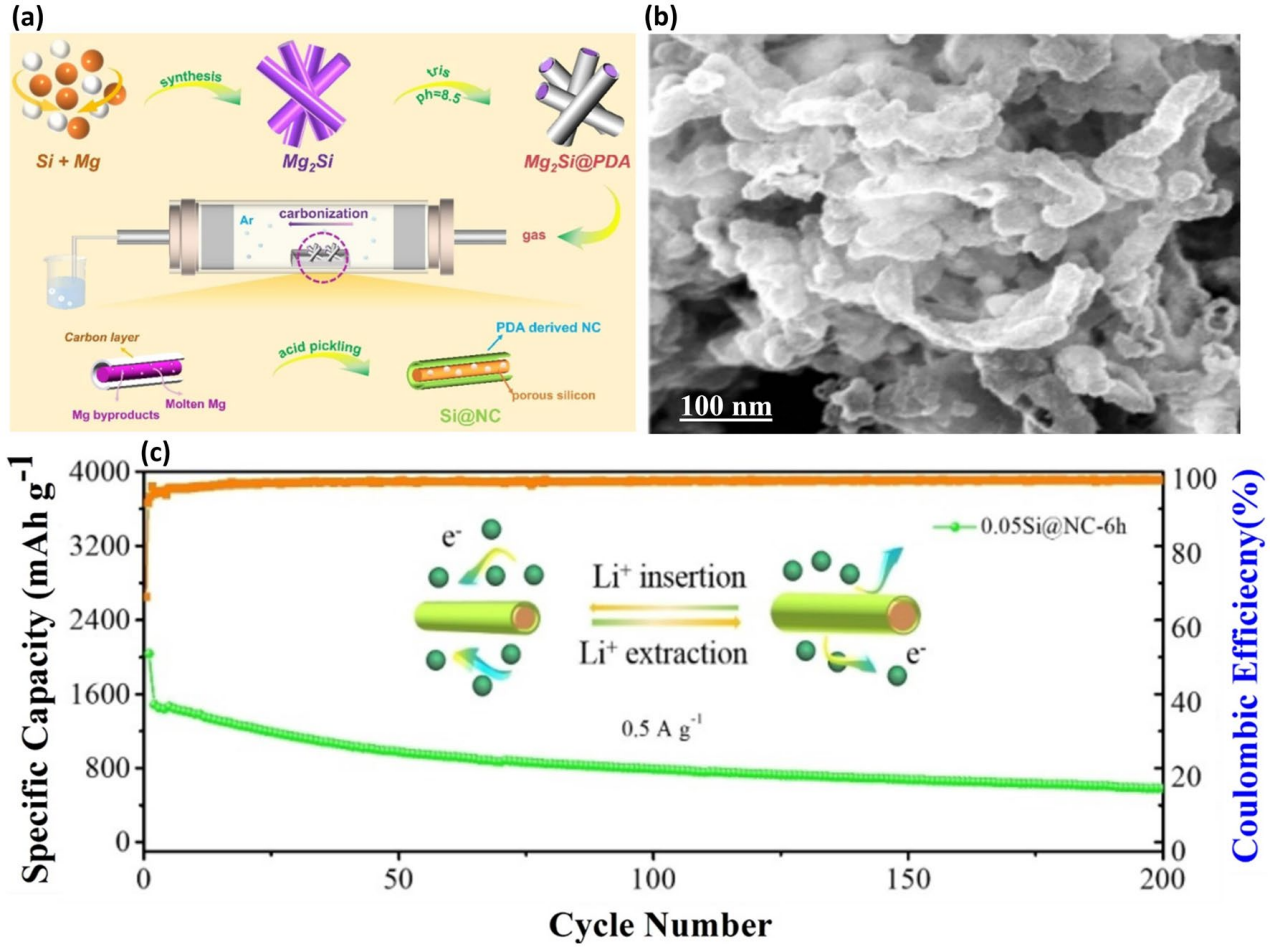
Synthesis and Performance of Si@NC Nanotube Composite—**a** Schematic illustration of the synthesis process for the Si@NC nanotube composite, detailing the steps involved in creating this advanced material. **b** SEM image showcases the Si@NC nanotube, highlighting its unique morphology and structure. **c** Long-term cycling capability of the Si@NC nanotube composite, evidencing its robustness and potential for use in energy storage applications. Reproduced with permission from Ref. [97]. Copyright 2022, Wiley Online Library

Si nanowires (SiNWs) and Si nanotubes (SiNTs) are prominent 1D structures in LIBs due to their unique attributes: Firstly, they possess the capability to mitigate mechanical strain during volume changes in the radial direction, preventing material pulverization. Secondly, they facilitate their facilitation of efficient electron transit, ensuring rapid charge transfer and, consequently, enhanced electrochemical performance. Significant efforts have been devoted to developing 1D-structured Si anodes using various innovative strategies. In 2008, Chan’s group [17] synthesized Si nanowires with a diameter of 90 nm utilizing a CVD. These nanowires exhibited commendable CE of 73% and improved cycle life, attributed to their well-structured nature accommodating substantial volume changes. Zhao et al. [97] introduced a novel 1D tubular silicon-nitrogen-doped carbon composite (Si@NC) with a core–shell structure, utilizing silicon magnesium alloy and polydopamine as a template and precursor (Fig. 8a). The morphology of the Si@NC composite was further analyzed using SEM (Fig. 8b), revealing that the diameter of silicon particles is approximately ~ 100 nm. The Si@NC composite demonstrates a remarkable specific capacity and ultrafast redox kinetics, exhibiting outstanding cycling stability with a fine capacity of 583.6 mAh g^−1^ at 0.5 A g^−1^ over 200 cycles (Fig. 8c). The engineered nanotube structure and silicon confined in nitrogen-doped carbon effectively alleviate volume expansion and confer superior stability.

Chen et al. [98] pioneered a novel self-supporting electrode with high mass loading capacity and enduring cycle life. It was based on carbon-coated SiNWs grown in situ on highly conductive, flexible carbon fabric substrates via a nickel-catalyzed one-pot atmospheric CVD technique. The superior quality of these carbon-coated SiNWs resulted in an impressive reversible specific capacity of 3500 mAh g^–1^ at 100 mA g^–1^. Beyond NWs, NTs represent another potential 1D configuration for Si anode materials. Song et al. [99] fabricated SiNT arrays on a stainless-steel substrate, utilizing a synthesis method combining templating and CVD. This structure demonstrated an admirable electrochemical performance of 2500 mAh g^−1^ after 50 cycles, attributed to the interspersed vacuum spaces between successive NTs. Wu and his team [100] designed a distinctive double-walled Si–SiO_*x*_ nanotube (DWSiNT) anode, featuring an active Si inner wall and a protective SiO_*x*_ outer layer. As an anode for LIBs, it maintained 94% of its original capacity after 500 cycles, with its structural design playing a pivotal role in this elevated electrochemical performance.

In 1D Si-based anode materials, the structural attributes of Si nanowires (SiNWs) and Si nanotubes (SiNTs) play a crucial role. Their elongated, one-dimensional form allows for efficient accommodation of volumetric expansion during the lithiation/delithiation processes, preventing material pulverization. Furthermore, the 1D structure facilitates rapid electron transport, significantly enhancing charge transfer and electrochemical performance. These unique properties, combined with advanced fabrication techniques like CVD and MACE, lead to controlled dimensions and improved electrochemical characteristics, thereby addressing the challenges associated with the use of Si in LIBs.

In summary, the exploration of one-dimensional (1D) Si-based anode materials has marked a significant milestone in enhancing the electrochemical performance of LIBs. Both Si nanowires (SiNWs) and Si nanotubes (SiNTs) have demonstrated exceptional promise owing to their structural attributes that enable effective accommodation of volumetric expansion and efficient electron transport, critical to high-performance LIBs.

Innovative fabrication techniques, such as CVD and metal-assisted chemical etching (MACE), have paved the way for the synthesis of highly ordered SiNWs and SiNTs with controlled dimensions and desirable electrochemical characteristics. The development of 1D structures like mesoporous Si nanowire arrays and double-walled Si–SiO_*x*_ nanotubes epitomizes the ongoing efforts to harness Si's high specific capacity while mitigating the adversities of volume expansion. Furthermore, the integration of carbon coatings and flexible substrates has emerged as a viable strategy to reinforce structural integrity and enhance electrical conductivity, factors paramount to sustaining long-term cycling stability.

Noteworthy are the reports of prolonged cycle lives and high specific capacities achieved by these 1D Si anodes, underscoring the effectiveness of these nanoengineered configurations. For instance, the pioneering work on self-supporting electrodes based on carbon-coated Si nanowires and the impressive performance of double-walled Si–SiO_*x*_ nanotube anodes exemplify the synergistic benefits of combining robust mechanical design with electrochemically active materials.

As we transition into the discussion of two-dimensional (2D) Si-based anode materials, it becomes evident that the lessons learned and successes garnered in the realm of 1D Si structures are invaluable. They not only serve as a testament to the advancements in nanomaterial synthesis and application but also set a solid foundation upon which further explorations into multidimensional Si-based materials for next-generation LIBs can be built.

#### 2D Si-Based Anode Materials

Si thin film, characterized as a two-dimensional structure, has garnered widespread attention as an anode material for LIBs. Its allure stems from its ability to mitigate the mechanical degradation challenges encountered during charge and discharge cycles. Greatz et al. [101] employed a 100-nm-thick amorphous Si thin film, revealing a specific capacity of 2000 mAh g^−1^ over 50 cycles, a remarkable achievement attributed to its nanoscale dimension. Chen’s team [102] applied a 275-nm-thick layer of amorphous Si on a Cu substrate for anode purposes, registering a reversible capacity of 2200 mAh g^−1^ after 500 cycles. Placke et al. [103] synthesized Mg-Si thin films by merging Si and Mg via magnetron sputtering, embedding the Si within an active magnesium-silicate matrix. This structure showcased a capacity of 1000 mAh g^−1^, retaining 96% of it over 400 cycles. Sun's team [104] utilized electrodeposition to produce a Si film on Ni foam, displaying an impressive reversible specific capacity exceeding 2800 mAh g^−1^ at a current of 360 mA g^−1^ through 80 cycles.

In 2D Si-Based Anode Materials, the planar structure of Si thin films plays a pivotal role. This structure offers a high surface-area-to-volume ratio, which is beneficial for reducing electron transport and lithium diffusion distances. This, in turn, minimizes polarization and enhances rate performance. Additionally, the thin-film form of Si provides effective management of mechanical stresses during the lithiation/delithiation cycles. This planar geometry allows for even distribution of stress and volume expansion, which is crucial for maintaining the structural integrity of the anode material over numerous cycles.

In summary, thin films emerge as a paramount solution to overcome Si's dual impediments: limited conductivity and pronounced volume expansion. Conventionally, the thickness of thin films spans from a fraction of a nanometer to several micrometers. The inherent advantages of this approach include higher average output voltage, reduced electrode mass, and extended cycle longevity compared to traditional LIBs. The structure of thin films, featuring with a superior surface-area-to-volume ratio, expedite lithiation and delithiation due to substantially minimized electron transport and lithium diffusion distances. Consequently, this curtails polarization, bolstering the rate performance. Moreover, this configuration adeptly manages the mechanical stresses arising from lithium-ion movement.

In retrospect, the realm of 2D Si-based anode materials has witnessed a transformative evolution, driving forward the efficacy and resilience of LIBs. Si thin films, epitomizing 2D structures, have captivated researchers due to their inherent ability to navigate the notorious mechanical deterioration prevalent during electrochemical cycling. This is predominantly facilitated by their planar geometry that significantly minimizes deleterious stress associated with lithiation and delithiation, thereby preserving structural integrity.

The breakthroughs chronicled within this domain exemplify meticulous material engineering and novel synthesis approaches. From employing amorphous Si layers of varying thicknesses to innovatively coupling Si with other elements such as magnesium, researchers have unfolded diverse strategies to enhance both the specific capacity and cycle stability of LIBs. For instance, the adoption of a multi-layered approach, leveraging active matrices like magnesium silicate, underscores the creative integration of materials aimed at stabilizing Si during battery operation.

Thin films’ characteristic high surface-area-to-volume ratio is pivotal, serving dual purposes: it shortens lithium ion and electron transport pathways, mitigating polarization and enhancing rate capability, and it proficiently accommodates volumetric expansions, a chronic setback with Si utilization in battery applications. This has culminated in demonstrable improvements in specific capacities, cycling life, and overall battery performance metrics.

Furthermore, the synthesis methods, ranging from electrodeposition to magnetron sputtering, reflect a sophisticated evolution of thin-film technology, reinforcing the versatility and adaptability of 2D Si-based anodes. This versatility extends beyond material composition to encompass advancements in structural design, including hybrid architectures that meld the benefits of Si with synergistic materials.

As we venture into the exploration of three-dimensional (3D) Si-based anode materials, the insights garnered from 2D structures are indispensable. They delineate a pathway rife with potential for overcoming the long-standing obstacles associated with Si anodes. Thus, the transition to 3D configurations is a logical progression, building on the foundational understanding established through the study of 2D Si anodes, and symbolizing a quest to further exploit Si's volumetric charge storage capacity without compromising structural and electrochemical stability.

#### 3D Si-Based Anode Materials

3D macroporous Si-based structures have attracted significant attention as anode materials for LIBs due to several reasons: (1) the microporosity accommodate volume variations during the lithiation/delithiation process without compromising the structure or integrity of the material; (2) their continuous network of electrode materials enhances electrical conductivity; and (3) the macropores, spanning hundreds of nanometers, facilitate rapid liquid-phase Li-ion transport, reducing polarization and boosting electrochemical performance. Bang et al. [105] developed 3D macroporous Si materials by integrating electroless metal deposition through a galvanic displacement reaction with a metal-assisted chemical etching method, utilizing commercially available bulk Si powders. This material exhibited a reversible capacity of 2050 mAh g^−1^. Yang et al. [106] fabricated a 3D macroporous Si with a consistently interconnected architecture via magnesiothermic reduction. They achieved adjustable morphological control of 0D hollow nanospheres by simply altering reaction conditions. As anode materials in LIBs, the void space in both architectures efficiently accommodates the significant volume changes linked to lithium insertion and extraction. Due to the robustness of the interconnected porous structure, the 3D Si@C electrode displayed superior electrochemical characteristics, including a reversible capacity of 1058 mAh g^−1^ after 800 cycles and a capacity retention rate of 91%.

**Fig. 9:**
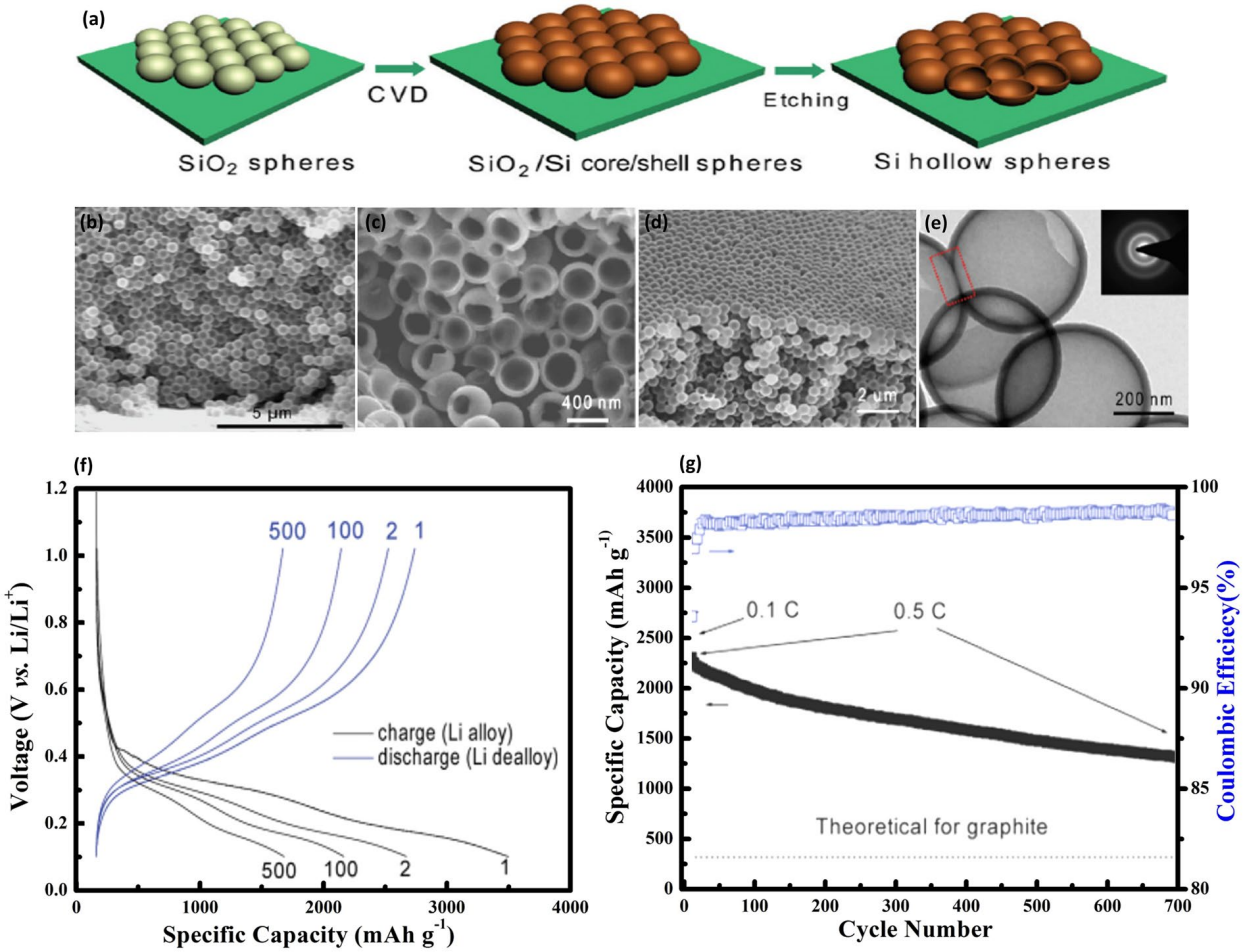
Fabrication and Analysis of Hollow Si Nanospheres for Energy Storage Applications—**a** Synthesis process for hollow Si spheres, beginning with the coating of Si nanoparticles on a stainless-steel substrate, followed by CVD deposition of Si, and concluding with the removal of the SiO_2_ core via HF etching to achieve the hollow structure. **b** Typical cross-sectional SEM image of the hollow Si nanospheres, illustrating the uniformity and integrity of the hollow structures. **c** SEM side view of the same sample, offering a different perspective on the morphology. **d** SEM image of hollow Si nanospheres that have been scraped open with a sharp razor blade, revealing the interior empty space and confirming the hollow nature. **e** TEM image of interconnected hollow Si spheres, highlighting their potential for enhanced electrochemical performance through structural connectivity. **f** GCD profiles at a rate of 0.5 C, noting that the first cycle was performed at 0.1 C to establish baseline performance. **g** Compares the reversible Li discharge capacity and Coulombic efficiency (CE) of the hollow Si nanospheres against the theoretical capacity of graphite, demonstrating the superior performance and potential of hollow Si nanospheres in energy storage devices. Reproduced with permission from Ref. [108]. Copyright 2011, American Chemical Society

Liu and colleagues [107] utilized 3D porous silica derived from natural reed leaves as precursors, subsequently producing a 3D ultra-porous SiC hierarchical structure through magnesiothermic reduction combined with a carbon coating method. This resulting architecture adeptly buffers considerable volume alterations and supports both electron and ion transport, achieving 420 mAh g^−1^ after 4000 cycles. Yao’s team [108] adeptly created interconnected, hollow Si nanospheres by using silica nanoparticles as templates. This process involved depositing Si layers on silica particles via CVD and then etching away the silica with HF to achieve hollow structures, depicted in Fig. 9a. The cross-sectional and side-view SEM images (Fig. 9b, c) showcase an orderly arrangement of the interconnected hollow Si spheres, revealing their substantial wall thickness and uniformity. Figure 9d exhibits a closer look at both the inner and outer surfaces of the hollow spheres, confirming their interconnectivity via shared shells, which is essential for maintaining structural resilience and enhancing electrochemical performance. The TEM image in Fig. 9e further confirms the hollow nature of the spheres and details their size and the amorphous quality of the Si material. Figure 9f exhibits the GCD profiles, illustrating impressive capacity retention and stability. Figure 9g shows the plots of the reversible Li discharge capacity and CE over numerous cycles, evidencing high efficiency and sustained capacity over 700 cycles-a significant improvement over conventional graphite anodes. Yu et al. [109] employed a silver mirror reaction to fabricate silver-coated 3D macroporous Si featuring consistent macropores around 200 nm in diameter, boasting a discharge capacity of 3585 mAh g^−1^ and an ICE of 81%.

The unique properties of 3D macroporous Si structures are central to their effectiveness as anode materials in LIBs. These structures provide substantial void space to accommodate the significant volume changes associated with lithium insertion and extraction, thus maintaining structural integrity. Their interconnected porous network enhances electrical conductivity, crucial for rapid electron transport and efficient charge transfer. Furthermore, the macropores facilitate rapid liquid-phase Li-ion transport, reducing polarization and boosting electrochemical performance. These combined attributes enable 3D Si anodes to overcome the challenges of volumetric expansion and unstable electrochemical behavior, characteristic of traditional Si anodes.

Reflecting on the progress within the sphere of 3D Si-based anode materials, it’s evident that this arena marks a significant departure from conventional approaches, addressing some of the most persistent challenges faced by Si anodes in LIBs. The introduction of 3D macroporous structures represents a paradigm shift, strategically tackling Si’s inherent issues related to volumetric expansion and unstable electrochemical behavior during battery operation.

The research initiatives discussed highlight a multifaceted approach to material and structural engineering. Key methodologies employed in the synthesis of 3D Si anodes—such as magnesiothermic reduction, galvanic displacement reactions, and chemical vapor deposition—underscore the intricate processes involved in producing these complex architectures. These techniques have not only enabled the creation of robust and highly interconnected porous networks but also facilitated unprecedented control over the morphological properties of the materials, as seen in the deliberate fabrication of hollow nanospheres and ultra-porous hierarchical structures.

One of the standout attributes of 3D macroporous Si is its ability to accommodate substantial volume changes during lithiation/delithiation. This is made possible by the presence of macropores and the unique structural integrity of the 3D framework, which collectively contribute to maintaining electrochemical stability over extended cycles. The impressive performance metrics—including high reversible capacity, admirable rate capability, and long cycle life—are testaments to the advantages conferred by this innovative material design. Table 4 provides the cycle stabilities of various composites anodes comprised of different Si and carbon materials for LIBs.

**Table 4:** Cycle stabilities of various composites anodes comprised of different Si and carbon materials for LIBs

Si Anodes	Current density (A g^−1^)	Cycle number	Remaining capacity (mAh g^−1^)	Refs.
Si/meso-C	2	50	700	[111]
Si/void/C	0.1	80	980	[112]
Si/C	0.372	100	1500	[113]
Si/MWCNT	0.4	70	520	[114]
Si/CNTs	0.42	200	925	[115]
Si/graphene/graphite foam	0.4	100	370	[116]
Graphene/Si/C	0.3	100	902	[117]
Si NWs	0.26	100	2000	[118]
SiNTs	0.4	90	~ 800	[119]
C@SiNTs	0.84	200	2085	[120]
CNTs/SiNTs	1.7	250	800	[121]
C@Si@CNTs	0.294	60	2200	[122]
Si@C/rGO	2	600	1100	[123]
Si/C	0.5	300	1041	[15]
Si/rGO	4	100	1521	[124]
SI/C	0.188	120	1179	[125]
Si/C-CNTs	0.5	200	1585.9	[126]
Si/C	4.2	500	1145	[127]
Si/NSs@C	0.1	200	822	[128]
C/Si	4	500	1072.2	[129]

Moreover, these studies have illuminated the critical role of void spaces and continuous electron/ion transport pathways in these structures. The strategic integration of carbon coatings and other elements further enhances conductivity and structural resilience, directly impacting the anodes’ overall performance.

Beyond the advancements in 3D Si-based anode materials in half-cell configurations, it is crucial to consider their performance in full-cell setups to provide a more comprehensive evaluation. Full-cell studies offer insights into the practical applicability of these anodes in real-world battery systems. For instance, a study detailed in ‘*Nature Communications*’ [110] discusses the preparation and characterization of Silicon Fuzz@Graphene (SF@G). This research includes comprehensive electrochemical characterization in both half-cells and full-cells, emphasizing the importance of full-cell performance in addition to half-cell data. Such full-cell analyses are imperative in assessing the overall efficiency and feasibility of 3D Si-based anodes in commercial LIBs.

As we transition to discussing SiO_*x*_/C type composites and the incorporation of Si with non-carbonaceous materials, the insights garnered from 3D Si-based anodes are invaluable. They serve as a robust foundation for future explorations aimed at optimizing composite materials that can harness the full potential of Si while mitigating its drawbacks. The journey ahead in battery material innovation beckons, promising new synergies, especially in composite anode configurations, that could redefine performance benchmarks in next-generation LIBs.

### SiO_***x***_/C Type Composites and Incorporating Si with Non-Carbonaceous Materials

**Fig. 10:**
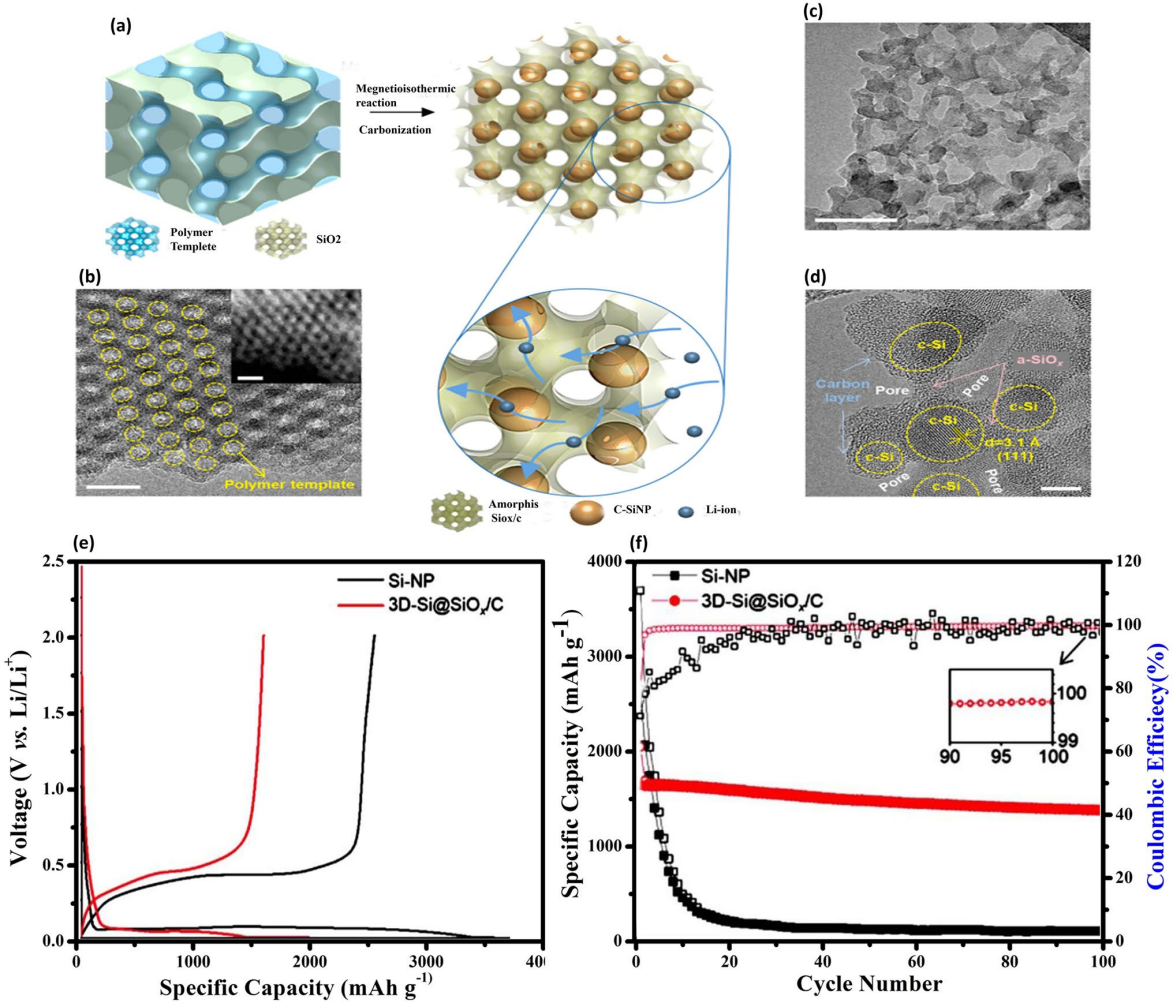
Development of a Gyroid 3D Network of Si@SiO_*x*_/C for Anode Applications—**a** Schematic illustration of the synthetic route to fabricate the 3D-Si@SiO_*x*_/C structure via one-pot magnetoisothermic reduction and carbonization of KIT-6, incorporating the polymer template for structure formation. **b** TEM image of the highly ordered double-gyroid KIT-6 including the polymer template, with a high-angle annular dark-field scanning TEM (HAADF-STEM) image shown in the inset, demonstrating the intricate gyroid structure and its uniformity. **c**, **d** TEM images of the 3D-Si@SiO_*x*_/C network, with **c** providing a broader view of the structure and **d** a high-resolution TEM (HRTEM) image highlighting the detailed crystalline structure. Scale bars are 20 nm for (**b**, including inset) and (**c**), and 5 nm for (**d**). **e** Galvanostatic charge–discharge (GCD) profiles of the 3D-Si@SiO_*x*_/C and Si nanoparticle (NP) anodes, showing the electrochemical performance comparison. **f** Cycling performances and Coulombic efficiencies (CEs) of the 3D-Si@SiO_*x*_/C and Si NP anodes at a current density of 200 mA g^−1^, indicating the enhanced durability and efficiency of the 3D-Si@SiO_*x*_/C structure. Reproduced with permission from Ref. [130]. Copyright 2019, American Chemical Society

SiO_x_ is a material considered attractive for LIBs due to its high specific capacity, excellent cyclic performance, and minimal volume fluctuations, approximately 160%, during the lithiation/delithiation process. These superior performances rise from the formidable strength of the Si–O bond and the formation of Li_2_O and lithium silicates, which counteract volume expansion. However, the formation of these oxides and silicates lowers the ICE of SiO_*x*_ materials, comprising electrochemical performance. A promising strategy is to encapsulate SiO_x_ within a carbon matrix. Carbon facilitates rapid electron and ion transport, supports the formation of the SEI layer, and can be readily sourced using various methods. Kim et al. [130] devised a creative method to construct a 3D gyroid-structured Si@SiO_*x*_/C nanoarchitecture (3D-Si@SiO_*x*_/C), which promotes enhanced ion/electron transport and superior thermal stability. They used hydrothermally synthesized double-gyroid highly ordered mesoporous silica, KIT-6, with a Pluronic P123 polymer template as the precursor, expecting to produce a Si-rich 3D network structure with precisely controlled porosity and Si formation. As illustrated in Fig. 10a, this precursor was reduced using magnesium (Mg) at high temperature under an argon atmosphere, followed by a thorough HCl aq. washing to remove residual Mg compounds, resulting in the 3D-Si@SiO_*x*_/C composite. These structures, originated from the meticulously ordered double-gyroid KIT-6 with the polymer template, were illustrated in Fig. 10b. Figure 10c, d demonstrates the embedded crystalline Si nanoparticles (c-Sis) within a mesoporous 3D SiO_*x*_/C network, where the carbon layers derived from the polymer templates. The GCD curves comparison at the first cycle between the 3D-Si@SiO_*x*_/C anode and SiNP anode revealed similar redox potentials, highlighting the dominant role of crystalline Si in the anode’s capacity (Fig. 10e). Moreover, the 3D-Si@SiO_*x*_/C anode demonstrated a significant reversible capacity with high ICE and impressive capacity retention over 100 cycles, as depicted in Fig. 10f, indicating its suitability for high-energy-density applications.

Guo et al. [131] presented a unique and straightforward approach to fabricate high-performance SiO_*x*_/C composites. These composites, with a graphite-like structure, enable SiO_*x*_ particles to be efficiently distributed and anchored in carbon materials by reconfiguring the inherent structure of artificial graphite. Such multicomponent carbon materials are pivotal for addressing the challenges of SiO_*x*_-based anodes, especially in terms of forming a SEI, maintaining structural integrity of electrode materials, and enhancing the electrode’s electrical conductivity. The resulting SiO_*x*_/C anodes display high reversible capacities (645 mAh g^−1^), impressive cycling stability (around 90% capacity retention after 500 cycles), and superior rate capabilities. Cui’s group [132] derived a SiO_*x*_/C composite from rice husk using aluminoisothermal reduction, achieving a robust current density of 1230 mAh g^−1^ at 100 mA g^−1^. Similarly, Gao et al. [133] efficiently produced SiO_*x*_/C composite materials from agricultural rice husk waste. By subjecting rice husks to heat treatment at 900 °C in an argon/hydrogen atmosphere, they transformed them directly into a SiO_*x*_/C composite, which consists of SiO_*x*_ embedded within an amorphous carbon matrix. After 100 cycles, a stable reversible capacity of approximately 600 mAh g^−1^ was observed at a current density of 100 mA g^−1^. The exceptional performance of the SiO_*x*_/C composite anode, when compared to other agricultural byproduct-derived carbon materials, is attributed presence of low valence Si.

Jiang et al. [134] produced Si-SiO_*x*_@C through the direct pyrolysis of poly(methyl methacrylate) PMMA on the Si surface, resulting in a commendable capacity of 1030 mAh g^−1^ and a CE of 99.5% after 500 cycles. Lee’s group [135] created a Si-SiO_*x*_@C composite by uniformly embedding Si nanodomains in a SiO_*x*_ matrix topped with a carbon surface layer. This composite demonstrated a specific capacity of 1561 mAh g^−1^ at 0.06 C and an ICE of 80%. Lv et al. [136] synthesized an SiO_*x*_/C composite by mixing a silica solution with sucrose. After ball milling and pyrolysis, the resultant SiO_*x*_/C exhibited an 820 mAh g^−1^ capacity at 100 mA g^−1^ and a 68.8% ICE. Ma et al. [137] fashioned a Si/SiO_*x*_@NC composite using magnetioisothermic reduction, encapsulating Si and SiO_*x*_ in nitrogen-doped carbon. After 100 cycles, this composite showed a capacity of 702 mAh g^−1^.

The blending of Si with carbon (Si/C) has emerged as a promising approach to enhance the electrochemical performance of Si-based anode materials in LIBs, especially because carbon's mechanical strength is not adequate to handle the volume expansion of the Si-based anode. Metals and metal oxides, due to their inherent mechanical robustness, have garnered increased attention as potential materials for Si-based systems. Bai et al. [138] designed a Si@TiO_2_ core–shell structure with a 3 nm thickness, displaying a reversible capacity of 1580 mAh g^−1^ after 50 cycles.

**Fig. 11:**
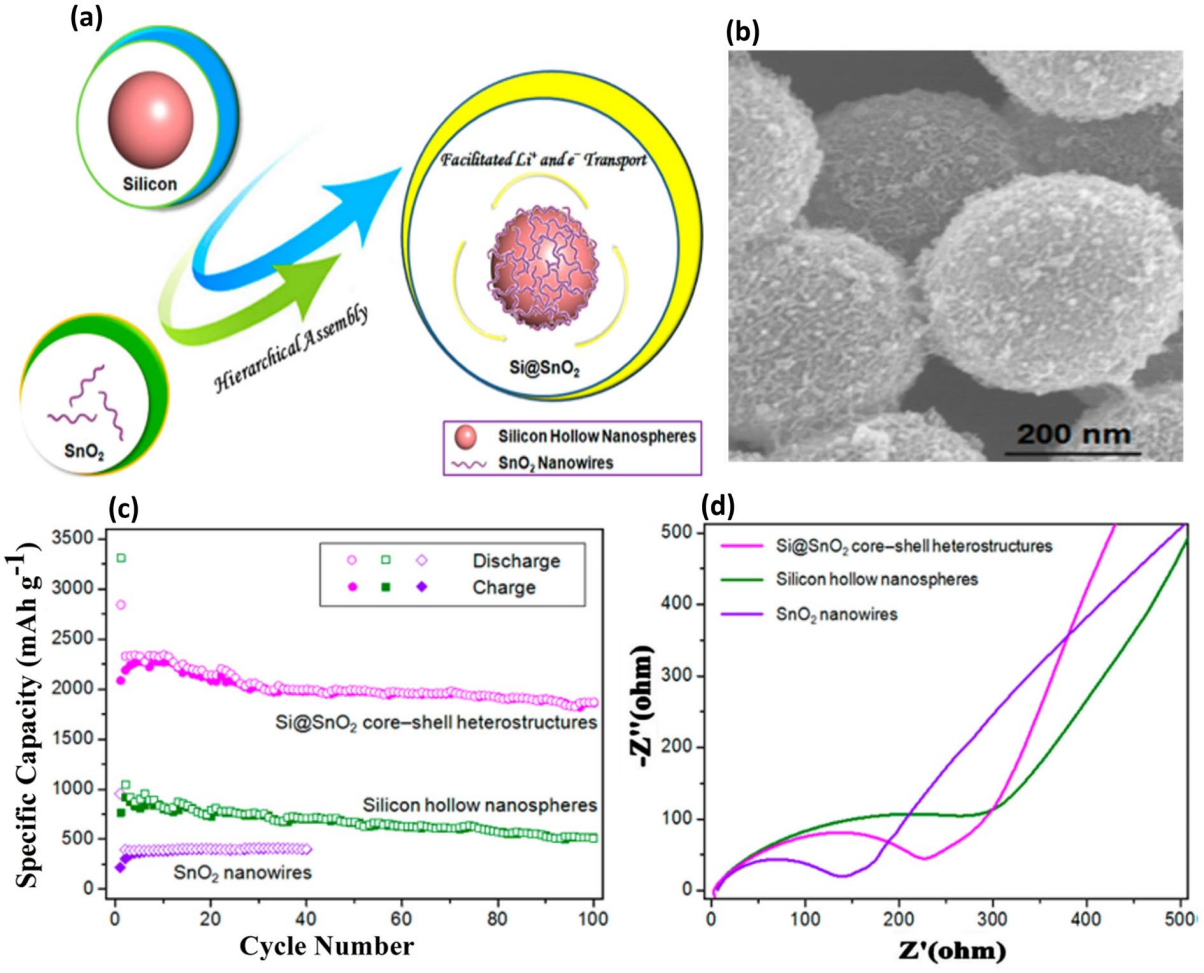
Formation and Evaluation of Si@SnO_2_ Core–Shell Heterostructures for Lithium-Ion Batteries—**a** Illustrates the process in which Si hollow nanospheres and SnO_2_ nanowires undergo sonication in tetrahydrofuran (THF), resulting in their spontaneous assembly through van der Waals interactions to form Si@SnO_2_ core–shell heterostructures. This combination leverages the properties of both materials to enhance lithium and electron transport. **b** SEM images provide a detailed view of the Si@SnO_2_ core–shell structures, showcasing the uniformity and quality of the heterostructures. **c** Cycle behaviors of Si@SnO_2_ core–shell heterostructures, Si hollow nanospheres, and SnO_2_ nanowires at a current density of 500 mA g^−1^, highlighting the superior cycling stability of the core–shell structures. **d** Nyquist plots illustrate the electrochemical impedance of the Si@SnO_2_ core–shell heterostructures, Si hollow nanospheres, and SnO_2_ nanowires, offering insights into the improved ionic and electronic conductivities of the core–shell configuration. Reproduced with permission from Ref. [139]. Copyright 2016, American Chemical Society

Owing to the high conductivity of SnO_2_, Si@SnO_2_ core–shell heterostructures were constructed by assembling SnO_2_ nanowires on the surface of hollow Si nanospheres via van der Waals interactions [139], in which the resulting conductive shell promotes rapid electrons and Li^+^ transport (Fig. 11a). Notably, this van der Waals interaction-based self-assembly is an innovative approach suitable for many 2D crystals self-assembly, each characterized by extensive surface areas and heightened surface energies. SEM images in Fig. 11b depicted the Si@SnO_2_ core–shell heterostructures, in which SnO_2_ nanowires were uniformly coated on these nanospheres with the diameter of around 300 nm. Figure 11c displays the remarkable cycling performance of the Si@SnO_2_ core–shell composites applied as LIB anode, in which the specific capacity of Si@SnO_2_ core–shell heterostructures was much higher than that of Si hollow nanospheres and SnO_2_ nanowires at a current density of 500 mA g^−1^. Moreover, EIS analyses of the Nyquist plots for these materials elucidated the conductivity of Si nanospheres was improved once SnO_2_ nanowires decorated on their surface (Fig. 11d), attributing the overall enhancement of the electrochemical performance. Yang et al. [140] synthesized Si@a-TiO_2_ using a straightforward solution-gel method, with Si nanoparticles as the core and a 3 nm thick amorphous TiO_2_ (a-TiO_2_) as the shell. After 200 cycles, Si@a-TiO_2_ exhibited a capacity of 1720 mAh g^−1^ at a current density of 420 mA g^−1^. Ma et al. [141] synthesized SnO_2_@Si via SiO_2_ etching, unveiling a capacity of 1030 mAh cm^−3^ over 500 cycles. A Si@Nb_2_O_5_ core–shell was fabricated through a simple solvothermal method [142], displaying a reversible capacity of 2256 mAh g^−1^ after 80 cycles.

The effectiveness of SiO_*x*_/C composites in LIBs stems from several key mechanisms. SiO_*x*_, with its high specific capacity and reduced volume fluctuation, forms Li_2_O and lithium silicates during operation, counteracting volume expansion. The carbon matrix in these composites enhances electron and ion transport, supporting SEI layer formation and maintaining structural integrity. Non-carbonaceous materials, such as metals and metal oxides, add mechanical robustness and stability, allowing for efficient electron and Li^+^ transport. These combined factors significantly boost the electrochemical performance of Si-based anode materials, addressing common challenges such as limited conductivity and volume expansion.

The exploration of SiO_*x*_ and its composites with carbon and non-carbonaceous materials make a significant advancement in the pursuit of high-performance anode materials for LIBs. This research thrust is driven by the remarkable electrochemical characteristics of SiO_*x*_, particularly its impressive specific capacity, cyclic resilience, and controlled volume expansion during battery operation. These advantages, however, do not come without challenges, as the intrinsic formation of Li_2_O and lithium silicates, though beneficial for addressing volume expansion, inherently lowers the ICE of SiO_*x*_-based materials, necessitating innovative material design strategies.

The encapsulation of SiO_*x*_ within carbonaceous matrices has emerged as a particularly effective method to overcome these limitations. Highlighted studies demonstrate that this approach not only enhances electron and ion transport, leveraging carbon's conductive properties, but also facilities the formation of a SEI layer. This SEI layer is a critical component for the anode’s long-term stability and performance. Notably, the innovative methodologies employed in the synthesis of these composites, ranging from one-pot synthesis techniques to the use of bio-derived materials like rice husk, underscore the diverse pathways available for the optimization of SiO_*x*_/C composites.

Furthermore, the realm of non-carbonaceous materials paired with Si, including various metals and metal oxides, unveils another frontier in anode enhancement. The synthesis of core–shell structures, where Si serves as the core surrounded by different metal oxides, presents a strategy that couples the high capacity of Si with the structural stability provided by the metal oxides. These configurations have demonstrated exceptional reversible capacities and admirable cycle stability, confirming the promise of this research direction. Table 5 gives the electrochemical capabilities of composites of Si with other materials as anode for LIBs.

**Table 5:** Electrochemical capabilities of Si with other materials as anode for LIBs

Si Anodes	Current density (A g^−1^)	Cycle number	Remaining capacity (mAh g^−1^)	Refs.
Si/Ag	0.84	100	1163	[109]
Si/Cu	2.1	60	2002	[143]
Si/ZnO	0.84	210	1500	[144]
SiNPs/V_2_O_5_	2.1	50	932	[145]
SiNPs/LIPON	2.1	100	1050	[146]
SiNPs/GR/Li_7_P_3_S_11_	4.2	500	1332	[147]
SiNPs/GR/Li_4_SiO_4_	0.21	40	1893	[148]
P_0.5%_ Si-Cu	0.1	60	1048	[149]
Si@LPO@void@FC	1	500	569	[150]
*β*-Si_3_N_4_/Si	5	100	480	[151]

As we advance to discussions on other factors influencing the performance of Si-based anode materials, it becomes increasingly clear that the journey toward optimal anode material for LIBs is multifaceted. It involves not only the chemical composition and structural design of the active materials but also a deeper understanding of the ancillary components and fabrication techniques. These elements collectively influence factors such as construction of artificial SEIs, prelithiation of the anode material and the choice of binder, which are integral to the holistic performance of the battery. The insights gained from studying SiO_*x*_/C composites and Si with non-carbonaceous materials play a crucial role in shaping future research trajectories, ultimately guiding us toward the next generation of high-efficiency, long-lasting LIBs.

## Other Factors Affecting the Performance of Si-Based Anode Materials

Integrating Si nanomaterials with conductive carbon, metals, and metal oxides has markedly improved the electrochemical performance of Si-based anode in LIBs through innovative structural engineering techniques. Despite these enhancements, the challenges of low CE and unstable SEI formation continue to impede the wide-scale adoption of Si-based anodes in commercial applications [152]. To address these issues, this section starts to investigate the engineered design of an artificial SEI (ASEI) specific to Si anodes. This engineered layer is tailored to obstruct electrolyte infiltration and decomposition and simultaneously promotes Li^+^ ion transport, thus reinforcing the Si anode interface and improving the anode’s cycling CE.

Subsequent to the ASEI discourse, the text delves into the synergistic effects of prelithiation processes aimed at augmenting initial CE and maintaining capacity integrity. Additionally, the development and efficacy of advanced binder systems are meticulously evaluated, discerning their pivotal role in reinforcing structural fidelity and electrochemical performance of Si-based anodes for LIBs.

### Artificial Solid Electrolyte Interphase for Si Anode

In the pursuit of enhanced performance and safety in LIBs, the SEI plays a crucial role. This passivation layer forms between the electrode and electrolyte during the electrochemical cycling of the battery, facilitating lithium-ion transport while impeding electron flow. It is typically composed of an intricate matrix of inorganic and organic species, including Li_2_CO_3_, LiF, Li_2_O, HCOLi, ROLi, and ROCO_2_Li, with the alkyl group (R) being solvent-dependent [153]. However, the naturally formed SEI is often brittle and heterogeneous, rendering it prone to cracking or delamination over the battery’s lifespan. This degradation not only exposes the anode to the electrolyte, instigating additional SEI growth and capacity fade but also raises safety concerns due to the potential for uneven lithium plating, which can lead to dendrite growth [154].

To overcome the deficiencies of the naturally forming SEI, particularly in high-capacity anodes like Si that experience extensive volume changes during alloying and dealloying cycles, the construction of artificial SEIs has been proposed as a strategic alternative [154]. These ASEIs, typically applied through ex situ processes, aim to provide a more robust and stable protective layer compared to the naturally formed SEI. Building upon these strategies, ASEIs can be constructed using three primary methods, each addressing specific aspects of SEI formation and stability:

#### Electrolyte Modulation

**Fig. 12:**
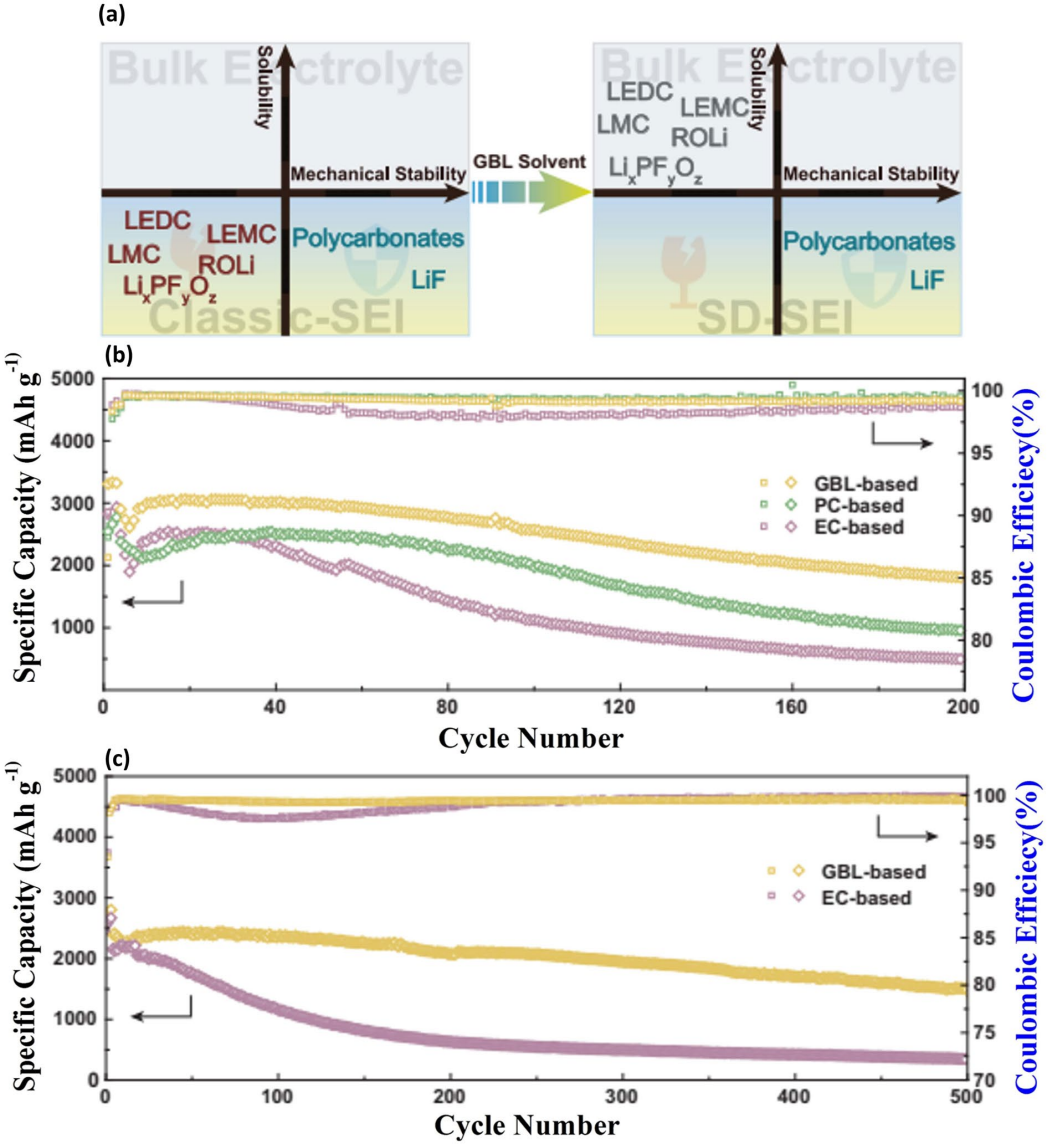
Solvent-Induced Selective Dissolution for SEI Optimization in Si Anodes—**a** Schematic representation of the solvent-induced selective dissolution process for solid electrolyte interphase (SEI) layers, highlighting how SEI components are differentiated based on their solubility in various solvents. This technique aims to refine SEI composition for improved anode performance. **b** Cycling performance and Coulombic efficiencies (CEs) of micron-sized Si anodes tested in different electrolytes at a charge/discharge rate of 0.2 C, where 1 C equals 3000 mA g^−1^, illustrating the impact of electrolyte choice on anode durability and efficiency. **c** Similar data for Si@C (silicon-carbon composite) anodes in varied electrolytes at 0.2 C, demonstrating the role of SEI optimization in enhancing the electrochemical performance of composite anodes. Reproduced with permission from Ref. [155]. Copyright 2023, Springer Nature

This method involves altering the composition of the electrolyte to promote the formation of a more robust and uniform SEI layer. For instance, the introduction of additives like gamma-butyrolactone in traditional electrolytes can selectively dissolve lower-modulus components of the SEI, resulting in a layer predominantly composed of lithium fluoride and polycarbonates. This method has shown to significantly enhance the mechanical properties of the SEI, as demonstrated in the work of Tian et al. [155]. The process is visually illustrated in Fig. 12a, where the selective dissolution strategy for in situ regulation of the SEI's mechanical properties is depicted. This novel approach has shown promising results, with raw micron-sized Si anodes retaining 87.5% capacity after 100 cycles at 0.5 C (1500 mA g^−1^, 25 °C), as shown in Fig. 12b. Furthermore, when applied to carbon-coated micron-sized Si anodes, the cycle life could be extended to more than 300 cycles, evident from the data in Fig. 12c.

#### Inorganic Material Deposition

This method entails depositing inorganic materials such as lithium phosphorus oxynitride (LiPON) onto Si particles to form a protective layer. LiPON and similar materials offer superior ionic conductivity and electrochemical stability, preventing electrolyte decomposition and mitigating volume expansion during cycling. Liang’s group [146] highlighted that a critical LiPON layer thickness of 40–50 nm is essential for optimal performance.

#### Organic Material Coating

This strategy involves coating Si particles with organic materials like citric acid, polyacrylic acid (PAA), or a carboxymethyl cellulose-citric acid (CMC/CA) composite. These coatings can improve the mechanical flexibility of the SEI and enhance its electrochemical stability. Patolsky et al. [156] demonstrated that a poly p-xylylene (parylene) coating can form a nanometrically thin, highly resilient SEI layer, significantly extending the lifespan of the anodes.

**Fig. 13:**
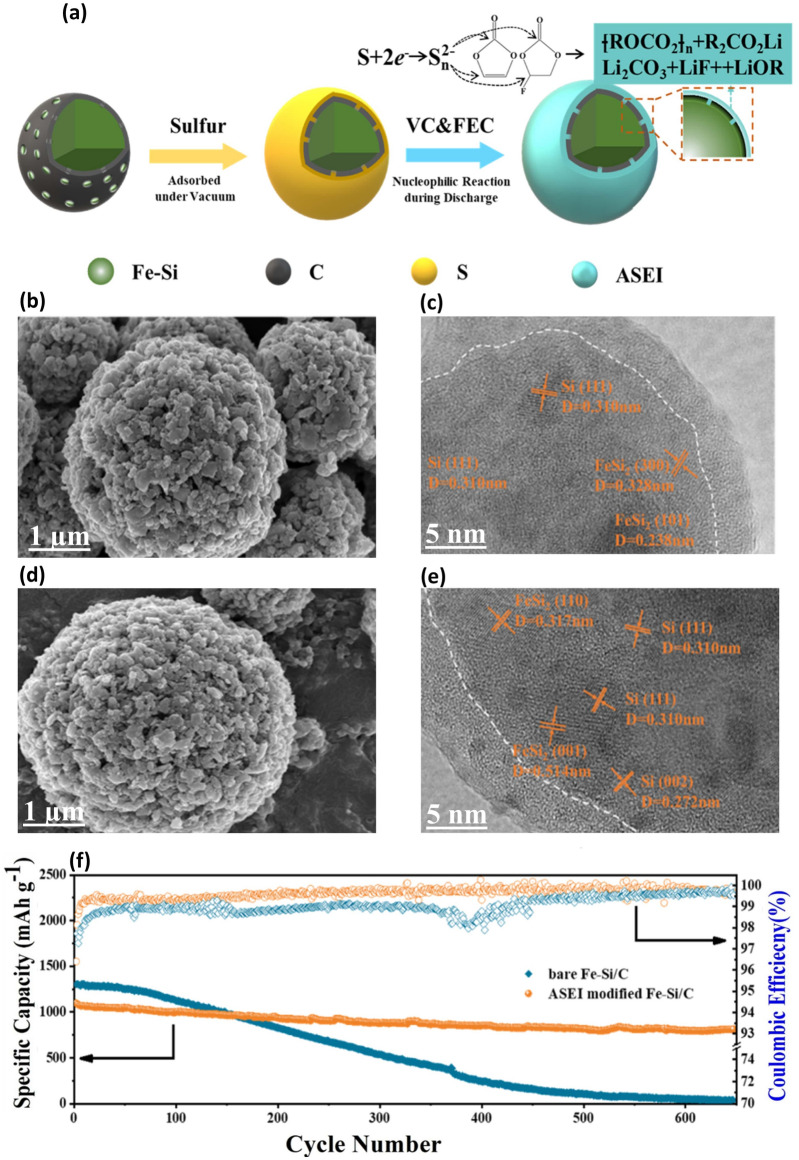
Fabrication and Characterization of ASEI-Modified FeSi/C Anode for Enhanced Cycling Stability—**a** Fabrication process for the ASEI-modified FeSi/C anode, detailing the steps involved in creating the advanced sulfur-adsorbed electrode interface. **b** SEM and **c** HRTEM images of the pristine FeSi/C composite exhibit the initial microstructure and crystallinity. **d** SEM and **e** HRTEM images post sulfur adsorption on the FeSi/C composite showcase modifications in the surface and structural properties aimed at improving electrochemical performance. **f** Cycling stability and Coulombic efficiencies (CEs) of the ASEI-modified FeSi/C composite at a current density of 500 mA g^−1^, highlighting its enhanced durability and efficiency in battery applications. Reproduced with permission from Ref. [157]. Copyright 2021, Springer Nature

The method developed by Wang et al. [157] for creating an ASEI on a ferrosilicon/carbon (FeSi/C) composite anode is delineated in Fig. 13a. The process commences with the adsorption of sulfur onto the FeSi/C composite via a vapor transfer method under vacuum. This sulfur-adsorbed composite is then processed into an electrode film and integrated into lithium coin cells with an electrolyte containing vinylene carbonate (VC) and fluoroethylene carbonate (FEC), where the ASEI layer in situ forms during the initial discharge phase. Further analysis using SEM, and TEM, was conducted on both untreated and sulfur-adsorbed FeSi/C composites. As depicted in Fig. 13b, the unmodified FeSi/C composite exhibits spherical aggregates, while the HRTEM image in Fig. 13c reveals a core–shell structure within the primary particles. Introduction of sulfur results in an enhanced external layer, as detailed in Fig. 13d, e.

The comparative cycling performance of the pristine and ASEI-modified FeSi/C anodes is presented in Fig. 13f. After initial activation at 100 mA g^−1^, the anodes were cycled at 500 mA g^−1^. The ASEI-modified anode demonstrated a remarkably stable capacity over 650 cycles, with a considerable retention of 74.2%. Additionally, the CE of the modified anode showed substantial improvement, stabilizing at 99.8% by the 200th cycle, a notable contrast to the pristine anode. Further investigation into the practicality of this technique revealed that a sulfur-adsorbed FeSi/C anode, with a high areal capacity of 4.1 mAh cm^−2^, maintained its performance when cycled at a current density of 1.0 mA cm^−2^, suggesting a promising approach to enhancing Si-based anodes in LIBs.

In summary, this section highlights the pivotal role of the SEI in LIBs, particularly concerning Si-based anodes where conventional SEIs prove insufficient due to their mechanical frailty and reactivity with the electrolyte. The reviewed research emphasizes the progress made in developing advanced ASEIs as a practical solution to the inherent challenges of Si anodes. ASEIs, with their tailored properties such as enhanced elasticity, toughness, and electrochemical stability, have demonstrated the potential to significantly improve the durability and safety of Si-based anodes. Innovations such as the incorporation of LiPON layers and the use of advanced materials like parylene have shown promising results in protecting anodes against electrolyte decomposition and handling the substantial volume changes during cycling. Particularly notable is the vapor transfer method used to create a sulfur-adsorbed ASEI on FeSi/C composites, which has yielded a remarkable increase in capacity retention and cycling stability. These advancements, verified through diverse imaging techniques and electrochemical performance tests, underscore the effectiveness of ASEIs in achieving more robust, reliable, and high-performance Si anodes, presenting a path forward for the commercial viability of high-capacity LIBs.

### Prelithiation of Anode Material

Prelithiation of anode materials plays a crucial role in enhancing the performance of LIBs, particularly when employing high-capacity anodes like silicon. This process is essential for compensating for the initial lithium loss incurred during the formation of the SEI in the first charging cycle, which becomes particularly crucial with lower quality electrolytes. The concept of prelithiation addresses critical challenges such as low CE and the need for improved electrical conductivity, thus making Si-based anodes more viable for practical applications.

Prelithiation techniques are diverse, each with its unique methodology and benefits. These techniques can be broadly categorized into direct contact methods (like lithium foil contact), chemical prelithiation (involving chemical reactions), and the use of stabilized lithium metal powder (SLMP). Each method offers different advantages in terms of ease of application, integration with existing battery manufacturing processes, and effectiveness in enhancing the anode's performance.

The necessity of prelithiation is underscored by several factors:

1. Addressing the low CE: Low CE is a significant barrier to practical application;
2. Enhancing the electrical conductivity: Prelithiation improves the electrical conductivity of the anode material, leading to enhanced cycle stability;
3. Utilizing a prelithiated anode coated with lithium: This approach results in a viable anode when paired with a lithium-free cathode.

To boost the performance of Si-based anodes in LIBs, a variety of prelithiation techniques has been explored, each offering unique advantages and applications. One such method involves lithium foil contact (LFC), where Yao et al. [158] employed an electrostatic-induced self-assembly method to produce Si nanoparticles encapsulated by graphene oxide nanoribbons (GONRs), enabling direct contact prelithiation. Prior to prelithiation, the composite exhibited discharge and charge capacities of 2721.3 and 2641.4 mAh g^−1^, respectively, with an ICE of 97.1%. Remarkably, the prelithiated electrode demonstrated exceptional cycling performance, maintaining a capacity of 1437.4 mAh g^−1^ after 300 cycles, with CEs of 97.9% and 98.6% in two respective cycles. Kim et al. [159] utilized a prelithiation technique, using vacuum thermal evaporation to uniformly predeposit lithium onto the Si-graphite anode, a process contributing to the ICE's increase from 80.4% to 89.6%.

Another effective prelithiation technique involves the use of stabilized lithium metal powder (SLMP). Notably, SLMP, with its larger specific surface area compared to lithium foil, forms a stable SEI layer when interacting with the electrolyte. This compensates for the irreversible capacity loss of the initial cycle, thereby increasing the energy density. Yao et al. [160] developed a unique free-standing flexible SiNPs-MWCNTs composite paper using ultrasonication and pressure filtration, initially exhibiting a capacity of 2298 mAh g^−1^ and a CE of 65%. Upon SLMP addition, the CE surged to an impressive 98%. Pan et al. [161] coated SLMP on a d-SiO/G/C anode surface, achieving an exceptional 98.5% ICE, highlighting the efficacy of SLMP in enhancing performance.

**Fig. 14:**
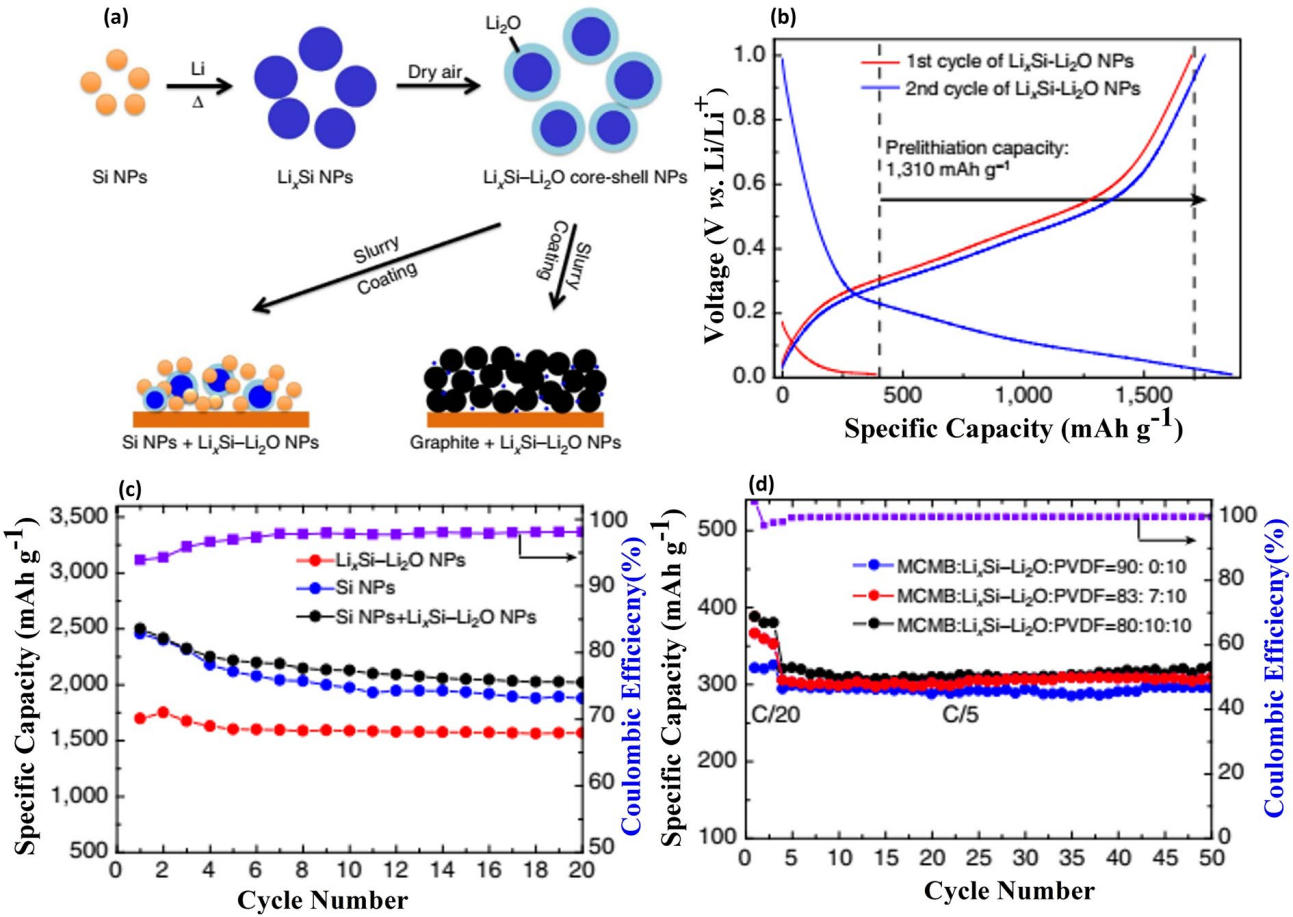
Interaction of Si NPs with Melted Li and Electrochemical Performance of Li_*x*_Si–Li_2_O Nanoparticles—**a** Schematic diagrams illustrate the reaction of silicon nanoparticles (Si NPs) with melted lithium (Li) to form lithium silicide (Li_*x*_Si) nanoparticles, depicting the initial step in creating advanced anode materials. **b** Galvanostatic charge–discharge (GCD) profiles for Li_*x*_Si–Li_2_O nanoparticles during the first and second cycles, indicating the electrochemical behavior and capacity retention. **c** Cycling performance of Li_*x*_Si–Li_2_O nanoparticles, Si NPs/Li_*x*_Si–Li_2_O composite, and control Si nanoparticles at a rate of C/20, with the purple line representing the Coulombic efficiency (CE) of the Si NPs/Li_*x*_Si–Li_2_O composite, demonstrating the enhanced performance of the composite material. **d** Cycling performance of mesocarbon microbead (MCMB)/Li_*x*_Si–Li_2_O composites with varying weight ratios, tested at C/20 for the first three cycles and C/5 for subsequent cycles (1 C = 0.372 A g^−1^, with capacity based on the total mass of active materials, including MCMB and Si in Li_*x*_Si-Li_2_O nanoparticles). The purple line indicates the CE of the MCMB/Li_*x*_Si–Li_2_O composite (80:10 by weight), highlighting the composite’s improved cycling stability. Reproduced with permission from Ref. [163]. Copyright 2017, Springer Nature

Chemical prelithiation represents another avenue for enhancing battery performance. Zhu et al. [162] demonstrated the production of selectively prelithiated Si@SiO_*x*_, boasting an ICE of 89.1% and a reversible capacity of 954 mAh g^−1^ at 30 mA g^−1^. Cui et al. [163] pioneered the development of Li_*x*_Si–Li_2_O core–shell nanoparticles through a one-step thermal alloying method. These nanoparticles presented significant promise for industrial battery assembly due to their ease of processing in a slurry and impressive capacity in ambient conditions, especially when shielded by a Li_2_O passivation shell. Prelithiation utilizing these nanoparticles has proven beneficial for both Si and graphite anodes, exhibiting high ICEs ranging from 94% to 100%. The incorporation of these nanoparticles enables the seamless integration of high-performance electrode materials into LIBs, as demonstrated in Fig. 14.

A myriad of prelithiation techniques, including LFC, SLMP, and chemical prelithiation, have been recently leveraged to alleviate lithium losses in LIBs, enhancing their electrochemical performance by amplifying ICE and rate performance. The LFC process involves submerging the active material (Si) in the electrolyte, in close proximity to the lithium metal foil. Upon applying pressure, a short circuit is initiated between the active material and the lithium foil, resulting in lithiation of the active material. This process effectively maintains reversible capacity while minimizing volume changes, resulting in commendable electrochemical performance. Conversely, SLMP represents a sophisticated prelithiation technique that seamlessly integrates with current battery manufacturing methods. With an approximate diameter of 20 nm, SLMP particles boast a specific surface area 4.5 times that of lithium foil, substantially reducing the electrode's local current density. This reduction diminishes the polarization of the lithium anode, thereby elevating its overall performance. Chemical prelithiation typically involves submerging the electrode in a lithium-rich solution. This chemically induced approach diminishes the initial irreversible capacity loss and enhances the material's stability by preventing direct interaction with lithium, thereby enhancing the stability and security of prelithiation. Ongoing research on additional prelithiation procedures aims to further refine the electrochemical performance of LIBs [164].

In conclusion, prelithiation of Si-based anode materials has emerged as a robust strategy to address inherent challenges associated with LIBs, particularly the notorious initial lithium loss during SEI formation. Various prelithiation techniques have demonstrated significant enhancements in the batteries' ICE and overall electrochemical performance. These advancements are not only promising in terms of elevating energy density but also pivotal in stabilizing anode materials against extensive volume changes during battery operation. However, optimizing the performance of Si-based anodes is not solely dependent on addressing lithium loss. Another critical component significantly influencing the structural integrity and efficiency of these high-capacity anode materials is the binder, a topic that will be explored in the subsequent section.

### Effect of Binder

Historically, poly(vinylidene fluoride) (PVdF) has been a binder of choice in many battery applications, valued for its compatibility with established manufacturing processes. However, PVdF struggles to accommodate the substantial volume changes characteristic of Si-based anodes, leading to reduced cycle life and stability.

#### Multifunctional Binders

Emerging developments in binder technology have introduced multifunctional binders, such as self-healing and conductive binders, to address these limitations:

- *Self-healing Binders*: These binders can recover from mechanical damages, ensuring the long-term integrity of the electrode. For example, a study by Wang et al. [165] demonstrated the application of a self-healing binder in a Si-based anode, showing improved cycling stability and mechanical resilience.
- *Conductive Binders*: Enhancing the electrical conductivity of the anode material, conductive binders contribute to enhanced cycle stability and overall battery performance. A representative study by Liu et al. [166] highlighted the effectiveness of a conductive binder in a Si-based anode, resulting in significant improvements in electrical performance and battery life.

Additionally, alternative binders like alginate [167, 168], carboxylic methyl cellulose (CMC) [169], and polyacrylic acid (PAA) [170] to address the shortcomings of traditional binders. Kovalenko et al. [171] employed alginate as a binder for a nano-Si anode, achieving a remarkable cyclic performance of 1700 mAh g^−1^ at 140 mA g^−1^. This achievement was attributed, in part, to the evenly distributed carboxyl groups within the alginate polymer chain, enhancing its interaction with Si and consequently improving anode stability during cycling. Wu et al. [172] explored various alginate-based binders (Al-alg, Ba-alg, Zn-alg, Ca-alg, and Mn-alg) with Si as the anode material. Among these, the Al-alg binder exhibited exceptional performance, reaching a capacity of 2100 mAh g^−1^ at 4200 mA g^−1^ after 300 cycles, while the Ba-alg binder retained a capacity of 840 mAh g^−1^ after 200 cycles. Another notable advancement came from Wang et al. [173], who improved the cyclic stability of a Si-based anode by introducing a self-healing polymer (SHP) binder. This SHP binder demonstrated superior electrochemical performance compared to PVdF, CMC, and alginate binders, owing to its mechanical and electrical self-healing properties. This nuanced advantage of SHP binders, especially in comparison to PVdF, CMC, and alginate, marks a significant leap forward, promising to address the enduring challenges associated with Si-based anodes.

**Fig. 15:**
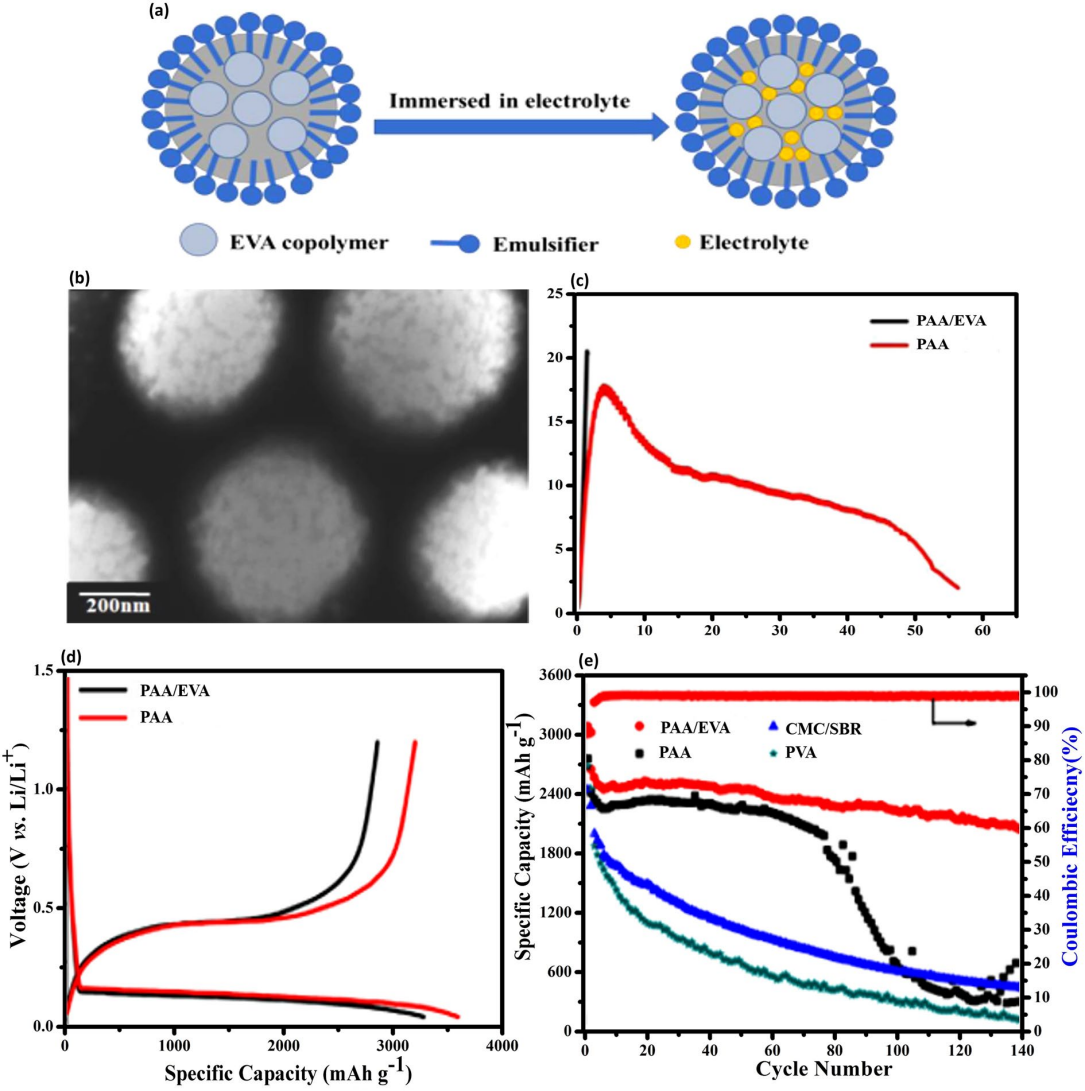
Evaluation of EVA Colloid in Electrolyte for Enhanced Anode Performance—**a** Depicts the schematic illustration of the ethylene–vinyl acetate (EVA) colloid structure immersion process in the electrolyte, highlighting the preparation steps for modifying the electrode’s surface. **b** A TEM image showcases the microstructure of EVA latex, providing insight into the colloidal configuration. **c** Tensile curves for polyacrylic acid (PAA) and PAA/EVA films are presented, comparing the mechanical properties and flexibility of the films. The electrochemical performance of porous Si anodes using PAA/EVA and PAA as binders is examined through **d** the initial charge–discharge profiles of electrodes at a current density of 50 mA g^−1^, demonstrating the impact of binder selection on electrode capacity and efficiency. **e** Reversible capacity and Coulombic efficiency (CE) of electrodes at a current density of 50 mA g^−1^ for the first two cycles and at 500 mA g^−1^ for subsequent cycles, indicating the durability and performance enhancement provided by the PAA/EVA binder combination. Reproduced with the permission from Ref. [174]. Copyright 2019, American Chemical Society

Han et al. [174] introduced a groundbreaking binder solution for porous Si anodes by combing poly(acrylic acid) (PAA) and poly-(ethylene-co-vinyl acetate) (EVA) latex (PAA/EVA). This binder effectively addressed electrode pulverization and electronic contact loss caused by volume fluctuations during charge and discharge cycles. PAA’s abundant carboxyl groups enhanced the cohesion between porous Si particles, while EVA’s inherent elasticity improved binder ductility. The high-ductility PAA/EVA binder accommodated Si volume changes, ensuring electrode integrity during cycling. The unique EVA latex structure, as seen in Fig. 15b, allowed electrolytes to infiltrate and populate voids (Fig. 15a), enhancing binder performance. Mechanical evaluations revealed PAA/EVA’s superior tensile properties, with a rupture threshold of 58%, attributed to EVA rubber cavitation and stress concentration effects (Fig. 15c).

Electrochemical assessments demonstrated PAA/EVA’s excellence, achieving specific capacities of 3578 and 3185 mAh g^−1^ at 50 mA g^−1^ current density during discharge and charge respectively (Fig. 15d). This outperformance resulted from the superior ductility and electrolyte retention of the PAA/EVA binder. PAA/EVA’s stable cycling behaviors, with 80% retention after 140 cycles (Fig. 15e), highlighted the synergistic effect of PAA and EVA. The adaptable EVA colloid shape prevented binder fracturing, supported pulverized Si, maintained electrode integrity, and slowed capacity fading by minimizing direct Si-electrolyte interactions. In contrast, the PAA electrode rapidly deteriorated, retaining only 26% capacity 100 cycles.

The evolution from traditional PVdF binders to cutting-edge multifunctional and composite binders reflects the ongoing advancements in battery materials science. While alginate and its derivatives marked a departure from the inadequacies of PVdF, the emergence of multifunctional binders like self-healing and conductive binders ushers in a new era for Si-based anodes in LIBs. These developments are crucial in accommodating the physical changes of Si, indicating potential pathways for future research in binder technology and, consequently, the performance benchmarks of Si-based anodes.

In our thorough review, we have navigated the intricate domains of Si-based anodes for LIBs, delving into the depths of Carbon-Si composites, nanostructured Si materials, SiO_*x*_/C composites, their integration with non-carbonaceous materials, and other crucial factors influencing anode performance. We have rigorously examined the evolving roles of ASEIs, the transformative impact of prelithiation techniques, and the revolutionary advancements in binder technology. These discussions have not only highlighted the scientific ingenuity behind these developments but also their practical implications in enhancing the efficiency and durability of Si-based anodes. To complement our extensive narrative, we present Table 6 through 9 at the culmination of our analysis. These tables synthesize our findings into a coherent overview of the scalability and commercial viability of advanced materials and technologies for LIBs, serving as a crystallized reflection of our exploration. Table 6 details Carbon-Si composites, highlighting potential enhancements in LIBs' energy density and stability. Table 7 delves into nanostructured Si anodes, examining the influence of size and shape on performance. Table 8 explores SiO_*x*_/C composites and non-carbonaceous integrations, showcasing strategies to overcome traditional Si anodes' limitations. Table 9 examines advancements in Si-based anodes, focusing on the role of ASEI, binders, and the crucial aspect of prelithiation of anode materials, emphasizing strategies for offsetting initial capacity loss and enhancing silicon anodes' lifecycle in commercial applications. Together, these tables underscore the balance between material innovation and commercial applicability in LIBs.

**Table 6:** Generalized carbon-Si composites for LIBs: evaluating scalability and commercial feasibility

Composite type	Strategy/composition	Description	Scalability potential	Commercial feasibility
1D Carbon-Si Composites	CNFs and CNTs Integration with Si	Utilization of CNFs and CNTs to construct conductive networks and host matrices for Si-based anode materials, handling volume changes during charging/discharging. Examples include C-L-SC, Si@CNTs, and Si/CNFs	High scalability due to established manufacturing techniques for CNFs/CNTs	Good, considering growing demand in energy storage technologies
2D Carbon-Si Composites	Graphene and MXene Integration with Si	Incorporation of graphene and MXenes with Si, resulting in structures like Si@G, Si@N-G, and MXene/Si@SiO_*x*_@C. Enhanced by the surface area and conductivity of graphene and the unique properties of MXenes	Moderate, challenges in large-scale production of quality graphene and MXenes	Promising, but dependent on cost reduction and production advancements
3D Carbon-Si Composites	Porous Carbon and Graphite Integration with Si	Development of composites like gigaporous carbon microspheres and Si/graphite/carbon (Si-G/C) composites, leveraging the structural benefits of 3D carbon materials	High, given the existing large-scale production for porous carbon and graphite	Very feasible, especially in markets demanding high-performance batteries

**Table 7:** Generalized nanostructured silicon anode materials: scalability and commercial prospects

Nanostructure type	Strategy/composition	Description	Scalability potential	Commercial feasibility
0D Si-based anode materials	Si nanoparticles and composites	Utilization of porous Si nanoparticles and composites, including Si@TiO_2_ and Si/Ti_2_O_3_/rGO. Techniques include electroless etching, self-assembly, CVD	Moderate, challenges in consistent quality production at scale	Emerging, hinges on integration with current battery manufacturing processes
1D Si-based anode materials	Si nanowires (SiNWs) and nanotubes (SiNTs)	Development of 1D structures like SiNWs and SiNTs using CVD and other methods. Examples include Si@NC, carbon-coated SiNWs	High for SiNWs with established methods; moderate for SiNTs due to complexity	Promising, particularly for high-end applications requiring advanced battery properties
2D Si-based anode materials	Si thin films	Application of Si thin films on substrates, e.g., amorphous Si on Cu. Techniques include electrodeposition and magnetron sputtering	Moderate, dependent on deposition technologies and material handling	Feasible, with potential in niche markets and specialty applications
3D Si-based anode materials	3D macroporous Si structures	Creation of 3D macroporous Si using methods like magnesiothermic reduction and galvanic displacement. Examples include Si@C electrodes and hollow Si nanospheres	High, especially with advancements in 3D material synthesis techniques	Good, subject to demonstration of long-term durability and cost-effectiveness

**Table 8:** Generalized SiO_*x*_/C composites and non-carbonaceous integrations: pathways to scalable and commercially viable LIB anodes

Material type	Strategy/composition	Description	Scalability potential	Commercial feasibility
SiO_*x*_/C composites	Encapsulation of SiO_*x*_ in Carbon	Utilization of carbon matrices to encapsulate SiO_*x*_, enhancing electron/ion transport and forming a stable SEI layer. Examples include 3D-Si@SiO_*x*_/C, SiO_*x*_/C from rice husk	High, leveraging existing carbon material production infrastructures	Very promising, especially if cost-effectiveness is achieved
Si with non-carbonaceous materials	Core–shell structures with metals/metal oxides	Development of core–shell structures combining Si with metals/metal oxides like TiO_2_, SnO_2_, Nb_2_O_5_. Techniques include CVD, solvothermal methods	Moderate, challenges in uniform core–shell structuring at scale	Emerging, with potential in high-performance battery sectors

**Table 9:** Generalized enhancements in Si-based anode materials: a focus on scalability and commercial application

Aspect	Strategy/Technique	Description	Scalability potential	Commercial feasibility
Artificial solid electrolyte interphase (ASEI) for Si anode	Engineered design of ASEI	Construction of artificial SEI layers using ex situ and in situ techniques to provide a robust protective layer on the anode	Moderate, requires precise control over layer formation	Promising, essential for high-capacity, long-life batteries
Prelithiation of anode material	Various prelithiation Techniques	Techniques like lithium foil contact (LFC), stabilized Lithium Metal Powder (SLMP), and chemical prelithiation to compensate for lithium loss	High, especially with advancements in lithiation technologies	Very feasible, can significantly enhance the market competitiveness of Si-based LIBs
Effect of binder	Use of advanced binders	Exploration of alternative binders (e.g., alginate, PAA, SHP) tailored for Si-based anodes to accommodate extensive volume changes	High, as alternative binders can be integrated into existing battery production lines	Very promising, especially for advanced batteries requiring high stability and performance

## Conclusion and Perspectives

Si-based anodes stand on the cusp of revolutionizing LIBs, offering a confluence of high theoretical capacity and environmental benignity. Despite these advantages, the journey toward their commercial realization grapples with significant obstacles, primarily stemming from their inherent volumetric expansion and associated electrochemical instabilities. This review has outlined various strategies developed to address these issues, highlighting the integration of Si with diverse carbonaceous and non-carbonaceous composites, the design of Si nanostructures, artificial SEI, as well as the utilization of innovative prelithiation approaches and functional composited binders. The included structural engineering plays a particularly crucial role, optimizing the Si-based anode's structure to avoid various issues and enhance battery performance through synergistic effects. This comprehensive exploration signifies the multidimensional advancements in Si-based anodes and underscores the importance of structural engineering in mitigating challenges, thereby elevating overall battery performance.

As we propel into a future where the demand for efficient energy storage systems escalates, the pivotal role of Si in LIBs becomes increasingly crucial. To this end, several prospective avenues require exploration and development:

1. *Advanced Composite Structures*: While the amalgamation of Si with carbon in 1D, 2D, and 3D configurations has shown remarkable improvements in conductivity and structural integrity, there is substantial scope for the development of novel composite structures. These could involve hybrid materials that leverage the benefits of carbon’s conductivity while introducing new elements that could further buffer the effects of Si’s expansion or enhance the electrode/electrolyte interface stability.
2. *Nanoengineering Solutions*: Future research should, however, pivot toward the scalable synthesis of these nanostructures with controlled morphologies and properties tailored for specific applications. Creating a library of standardized, high-performance nanoengineered Si particles could accelerate their integration into commercial battery systems.
3. *Innovative Binder Technologies*: The advent of self-healing polymer binders has opened a new chapter in addressing the mechanical stresses within Si-based anodes. Continuing this trajectory, the exploration of binders with programmable properties—such as stimuli-responsive capabilities, improved adhesion, or intrinsic conductivity—could further prolong the lifespan and performance of Si anodes. Functional composited binder is highly recommended for the synergistic effects.
4. *Understanding and Optimizing Prelithiation Strategies*: A deeper understanding of the lithiation process at a fundamental level is essential. Concurrently, developing efficient, scalable, and safe prelithiation techniques will be crucial in moving beyond the laboratory scale, pushing the boundaries of CE, and reducing the cost per unit energy of the overall battery system.
5. *System-Level Integration and Testing*: A holistic approach toward the integration of enhanced Si-based materials into full battery systems is imperative. This encompasses rigorous testing under real-world conditions, lifecycle analyses, and safety evaluations, which will necessitate collaborative efforts spanning academia, industry, and regulatory bodies.

In light of these prospects, the quest for harnessing Si’s full potential in LIBs represents a multidisciplinary challenge that extends beyond material science. It beckons a global research initiative that transcends the traditional boundaries of electrochemistry, engineering, and manufacturing processes. Only through such concerted efforts can we expedite the transition from experimental curiosities to commercial technologies that could power the future sustainably. The path forward, albeit laden with challenges, glimmers with the promise of a world where energy storage is no longer a limiting factor but a catalyst for innovation across sectors.
